# Lsd1 as a therapeutic target in Gfi1-activated medulloblastoma

**DOI:** 10.1038/s41467-018-08269-5

**Published:** 2019-01-18

**Authors:** Catherine Lee, Vasilisa A. Rudneva, Serap Erkek, Marc Zapatka, Lianne Q. Chau, Silvia K. Tacheva-Grigorova, Alexandra Garancher, Jessica M. Rusert, Ozlem Aksoy, Robin Lea, Helai P. Mohammad, Jianxun Wang, William A. Weiss, H. Leighton Grimes, Stefan M. Pfister, Paul A. Northcott, Robert J. Wechsler-Reya

**Affiliations:** 1 Tumor Initiation and Maintenance Program, NCI-Designated Cancer Center, Sanford Burnham Prebys Medical Discovery Institute, La Jolla, CA 92037 USA; 2 Biomedical Sciences Graduate Program, University of California San Diego, La Jolla, CA 92093 USA; 3 Department of Developmental Neurobiology, St. Jude Children’s Research Hospital, Memphis, TN 38105 USA; 4 Hopp Children’s Cancer Center at the NCT Heidelberg (KiTZ), Heidelberg, 69120 Germany; 5 Genome Biology Unit, European Molecular Biology Laboratory (EMBL), Heidelberg, 69117 Germany; 6 Division of Pediatric Neurooncology, German Cancer Consortium (DKTK), German Cancer Research Center (DKFZ), Heidelberg, 69120 Germany; 7 Division of Molecular Genetics, German Cancer Research Center (DKFZ), Heidelberg, 69120 Germany; 8 Department of Neurology, University of California, San Francisco, CA 94158 USA; 9 Cancer Epigenetics DPU, GlaxoSmithKline, Collegeville, PA 19426 USA; 10 College of Life Sciences, Beijing University of Chinese Medicine, Beijing, 100029 China; 11 Division of Immunobiology and Center for Systems Immunology, Cincinnati Children’s Hospital Medical Center, Cincinnati, OH 45229 USA; 12 Heidelberg University Hospital, Department of Pediatric Hematology and Oncology, Heidelberg, 69120 Germany

## Abstract

Drugs that modify the epigenome are powerful tools for treating cancer, but these drugs often have pleiotropic effects, and identifying patients who will benefit from them remains a major clinical challenge. Here we show that medulloblastomas driven by the transcription factor Gfi1 are exquisitely dependent on the enzyme lysine demethylase 1 (Kdm1a/Lsd1). We demonstrate that Lsd1 physically associates with Gfi1, and that these proteins cooperate to inhibit genes involved in neuronal commitment and differentiation. We also show that Lsd1 is essential for Gfi1-mediated transformation: Gfi1 proteins that cannot recruit Lsd1 are unable to drive tumorigenesis, and genetic ablation of Lsd1 markedly impairs tumor growth in vivo. Finally, pharmacological inhibitors of Lsd1 potently inhibit growth of Gfi1-driven tumors. These studies provide important insight into the mechanisms by which Gfi1 contributes to tumorigenesis, and identify Lsd1 inhibitors as promising therapeutic agents for Gfi1-driven medulloblastoma.

## Abstract (web summary)

Medulloblastoma is one of the most prevalent malignant brain tumors in children and has very poor prognosis. In this study, the authors show, using a mouse model of medulloblastoma, that Gfi1 promotes tumor growth by recruiting Lsd1, that this interaction inhibits genes involved in neuronal differentiation, and that Lsd1 may be a therapeutic target in Gfi1-activated tumors.

## Introduction

Medulloblastoma (MB) is one of the most prevalent malignant brain tumors in children. Current standard of care consists of surgical resection followed by cranio-spinal radiation and multi-agent chemotherapy. While survival rates have improved, one-third of patients still succumb to the disease and survivors often experience debilitating side effects from treatment. Molecular analysis has identified four major subgroups of MB: Wingless (WNT), Sonic hedgehog (SHH), Group 3, and Group 4^1^. Among these, Group 3 tumors have the worst outcomes, yet they remain poorly understood^2^.

Because the majority of Group 3 tumors exhibit overexpression or amplification of the *MYC* oncogene^3^, MYC is believed to be a key driver of Group 3 MB. However, studies by our group and others suggest that MYC alone is not sufficient to promote tumor growth^4,5^. To identify additional driver events, we employed an integrative genomics pipeline and found a series of spatially clustered, somatic structural variants (SVs) in Group 3 and Group 4 MBs that repositioned highly active enhancers close to the genes encoding growth factor independent 1 (*GFI1*) or growth factor independent 1B (*GFI1B*)^6^. These so-called “enhancer hijacking” events resulting in overexpression of GFI1 or GFI1B, contribute to ~15–20% of Group 3 and 10–15% of Group 4 MBs^6,7^. Functional experiments in mice demonstrated that overexpression of *Myc* in combination with either *Gfi1* or *Gfi1b* was sufficient to transform murine neural progenitors into brain tumors resembling human Group 3 MB^6^. Together, these data established *GFI1* and *GFI1B* as novel, prevalent drivers of Group 3 and Group 4 MB.

To determine if *GFI1* family proteins might represent therapeutic targets for MB, we sought to gain a deeper understanding of their role in tumorigenesis. Targeting oncogenes is likely to be effective only if these genes are required for continued tumor growth. Although our previous studies demonstrated that GFI1 proteins cooperate with MYC to initiate MB formation, their role in tumor maintenance remains unclear. Moreover, transcription factors such as GFI1 and GFI1B are typically difficult to target directly, but cofactors that are required for transcription factor function can often represent excellent therapeutic targets^8^. Thus, we have attempted to identify proteins that are necessary for the oncogenic effects of Gfi1.

Here, we show that Gfi1 expression is required for MB tumor maintenance and describe a critical role for the lysine demethylase Lsd1 in mediating its oncogenic effects in MB. Our studies also demonstrate the exciting potential of pharmacological inhibitors of Lsd1 for treating Gfi1-dependent MB. Given the poor prognosis and lack of treatments currently available for Group 3 MB, these findings have important implications for therapy.

## Results

### Gfi1 is required for tumor maintenance

**Fig. 1:**
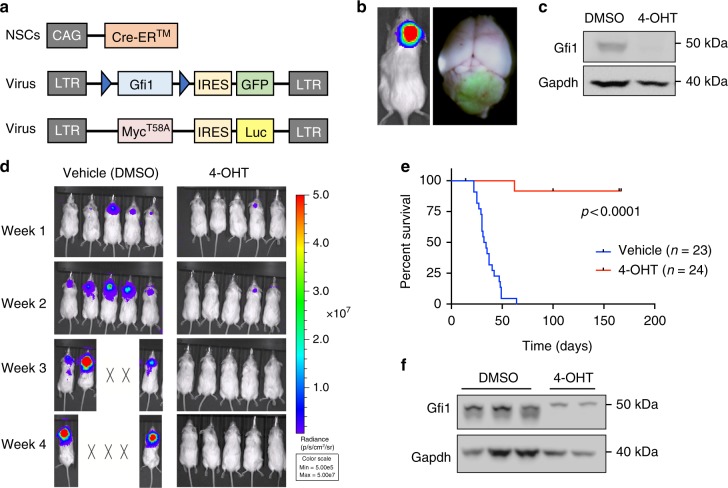
Gfi1 is required for tumor maintenance. **a** Constructs used to generate MG^flox^ tumors. NSCs from a CAG-CreER^TM^ mouse were transduced with viruses encoding Gfi1^flox^-IRES-GFP and Myc^T58A^-IRES-Luciferase. **b** Bioluminescence imaging and whole mount fluorescence image of representative MG^flox^ tumor. **c** Western blot for Gfi1 protein in MG^flox^ tumor cells treated overnight with vehicle control (DMSO) or 5 µM 4-hydroxytamoxifen (4-OHT). **d** Bioluminescence imaging of mice transplanted with cells treated with vehicle or 4-OHT. X’s denote animals euthanized before they could be imaged. **e** Survival curves from a representative experiment (control *n* = 23, 4-OHT *n* = 24). *p* Value < 0.0001 was determined by Log-rank (Mantel–Cox) test. **f** Western blot of Gfi1 protein in resulting MG tumors from vehicle and 4-OHT treatment groups

Our previous studies demonstrated that co-expression of *Myc* and *Gfi1* drives transformation of neural progenitors into MB cells^6^. Although these results indicate that *Gfi1* plays a role in tumor initiation, it is unknown whether it is required for continued tumor growth. To investigate this, we designed a conditional retroviral vector encoding Gfi1 flanked by loxp sites (Gfi1^flox^), which allows Gfi1 to be deleted by Cre recombinase (Fig. 1a). We isolated Prominin1 + neural progenitor cells^9^ from the cerebella of neonatal CAG-CreER^TM^ mice (which express tamoxifen-inducible Cre protein in all cells^10^) and infected them with viruses encoding Myc-IRES-luciferase and Gfi1^flox^-IRES-GFP. Orthotopic transplantation of infected cells into the cerebella of adult mice resulted in tumor formation within 5 weeks (Fig. 1b). The latency and penetrance of *Myc* + *Gfi1*^flox^ (MG^flox^) tumors was similar to that observed for *Myc* + *Gfi1*^WT^ (MG) tumors^6^ (Supplementary Fig. 1).

Treatment of MG^flox^ tumor cells with 4-hydroxytamoxifen (4-OHT) to activate CreER^TM^ caused a marked reduction in Gfi1 protein expression compared to cells treated with vehicle (DMSO) (Fig. 1c). Importantly, when cells were retransplanted into the cerebella of naïve mice, those that had been treated with vehicle gave rise to tumors within 4–5 weeks, whereas those that had been exposed to 4-OHT did not generate tumors in most recipients (Fig. 1d, e). Of 24 mice that received cells treated with 4-OHT, we observed only 2 cases where tumors developed, and Western blotting showed that these tumors still expressed Gfi1 protein (Fig. 1f). These studies demonstrate that continued expression of Gfi1 is necessary for maintaining tumor growth.

### Gfi1 recruits Lsd1

**Fig. 2:**
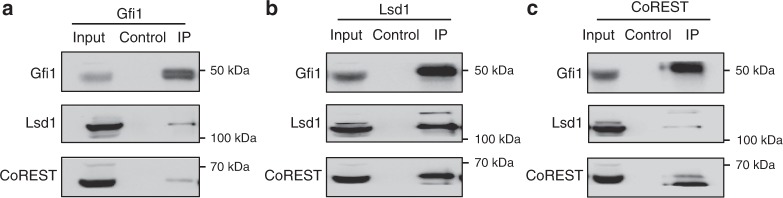
Gfi1 is associated with Lsd1. **a**–**c** Co-immunoprecipitations of **a** Gfi1, **b** Lsd1, and **c** CoREST were performed on MG tumor cells, and Gfi1, Lsd1, and CoREST protein levels were detected by Western blotting. Input represents 10% of total lysate before immunoprecipitation with experimental and isotype control antibodies

Given the importance of Gfi1 in MB initiation and maintenance, we sought to further understand the mechanisms by which Gfi1 promotes tumor growth. Studies in the hematopoietic system suggest that Gfi1/1b repress target genes via their interactions with cofactors, including Lsd1^11–14^, the corepressor CoREST^12,13,15^, and the histone deacetylases HDAC1 and HDAC2^16–18^. To determine whether these interactions are also involved in Gfi1-driven MB, we performed co-immunoprecipitation experiments on lysates from MG tumors. After immunoprecipitation of Gfi1, we detected interactions with Lsd1 and CoREST, but not HDAC1 or HDAC2, as determined by Western blotting (Fig. 2a; Supplementary Fig. 2). Immunoprecipitation of Lsd1 and CoREST yielded similar results, showing interactions with one another as well as with Gfi1 (Fig. 2b, c). Interestingly, the amount of Gfi1 protein detected after immunoprecipitation of Lsd1 or CoREST was similar to the amount detected after Gfi1 immunoprecipitation, suggesting that the majority of Gfi1 in the tumor cells complexes with Lsd1 and CoREST. In contrast, the amount of Lsd1 detected after immunoprecipitation of Gfi1 was only a small fraction of the total Lsd1, suggesting that Lsd1 also interacts with partners other than Gfi1. These data indicate that Gfi1 interacts with the epigenetic regulators Lsd1 and CoREST in Gfi1-driven MB.

### The SNAG domain is critical for Gfi1-driven tumorigenesis

**Fig. 3:**
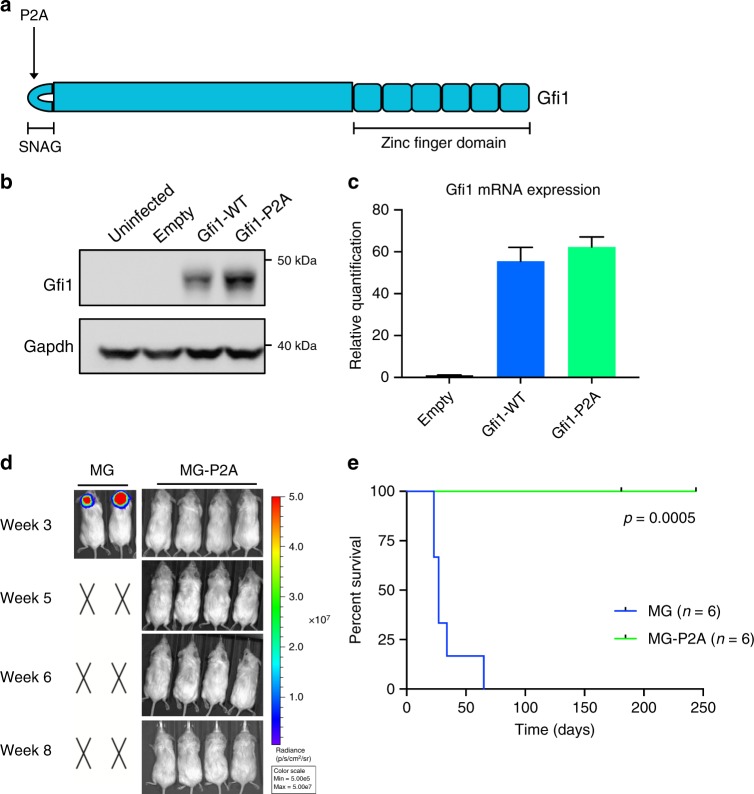
The SNAG domain is required for Gfi1-driven tumorigenesis. **a** Structure of Gfi1, illustrating the N-terminal SNAG domain and six C-terminal zinc fingers. Arrow denotes the proline to alanine point mutation of the SNAG mutant. **b** Western blot for Gfi1 protein levels in 293T cells transduced with empty vector, Gfi1-WT, or Gfi1-P2A. **c** qPCR for Gfi1 mRNA levels in NSCs transduced with empty vector, Gfi1-WT, or Gfi1-P2A. Data shown are from one representative experiment, where experiments were repeated in at least three biological replicates. Error bars represent 95% confidence intervals calculated using the sum of squares method. **d** Bioluminescence imaging of mice transplanted with NSCs that were co-infected with viruses encoding Myc and Gfi1-WT (MG) or Myc and Gfi1-P2A (MG-P2A) overnight. X’s denote animals euthanized before they could be imaged. **e** Survival curves comparing mice transplanted with MG or MG-P2A cells (MG *n* = 6, MG-P2A *n* = 6). *p* Value = 0.0005 was determined by Log-rank (Mantel–Cox) test

Gfi1 family proteins contain two highly conserved domains: a SNAG (Snail/Gfi1) domain at the N-terminus and six C_2_H_2_-type zinc fingers at the C-terminus^19^. The ability of Gfi1/1b to recruit and interact with cofactors such as Lsd1 and CoREST has been attributed to the SNAG domain^13,14,19^ (Fig. 3a). To determine the importance of this domain in MB pathogenesis, we utilized a *Gfi1* SNAG domain mutant with a proline to alanine change at amino acid 2 (*Gfi1*-P2A). This mutation has been shown previously to abrogate the function of the SNAG domain^19,20^; we confirmed this by co-immunoprecipitation, demonstrating that wild-type Gfi1 associates with Lsd1, but Gfi1-P2A does not (Supplementary Fig. 3). When overexpressed, the SNAG mutant still produced a full-length protein (Fig. 3b) and resulted in mRNA and protein levels comparable to those of wild-type *Gfi1* (Fig. 3b, c). We co-infected neural progenitors with *Myc* and the *Gfi1*-P2A mutant, transplanted them into NSG mice, and monitored animals for tumor growth. By 4–5 weeks, mice transplanted with cells carrying WT *Gfi1* had to be sacrificed (median survival = 27 days). In contrast, mice transplanted with cells expressing *Gfi1*-P2A were monitored for 7 months with no signs of tumor development (Fig. 3d, e). The stark difference in tumorigenic potential of the SNAG mutant strongly suggests that the ability of Gfi1 to recruit and interact with other proteins is essential for its oncogenic activity in MB.

### Genetic deletion of Lsd1 impairs growth of Gfi1-driven MB

**Fig. 4:**
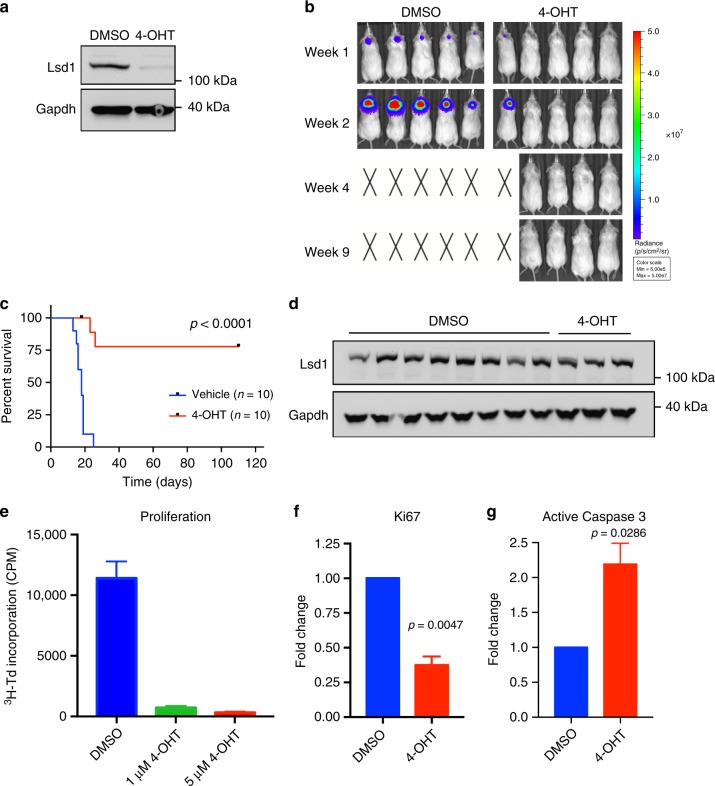
Genetic deletion of Lsd1 impairs growth of Gfi1-driven MB. **a** Western blot for Lsd1 protein in Lsd1-iKO tumor cells that were treated overnight with vehicle control (DMSO) or 5 µM 4-hydroxytamoxifen (4-OHT). **b** Bioluminescence imaging of mice transplanted with Lsd1-iKO cells treated with vehicle or 4-OHT. X’s denote animals euthanized before they could be imaged. **c** Survival curves from a representative experiment (control *n* = 10, 4-OHT *n* = 10). *p* Value < 0.0001 was determined by Log-rank (Mantel–Cox) test. **d** Western blot for Lsd1 protein in tumors from vehicle and 4-OHT treatment groups. **e** In vitro proliferation assay for Lsd1-iKO MG tumor cells treated with vehicle (DMSO), 1 or 5 µM 4-OHT. Proliferation was measured via ^3^H-thymidine incorporation at 48 h. Data are from a representative experiment and are plotted as the means of technical triplicate samples ± SEM. Experiments were repeated in at least three biological replicates. **f** Quantification of Ki67 staining in Lsd1-iKO MG cells after treatment with vehicle or 5 µM 4-OHT. *p* Value = 0.0047, *t* = 10.27, d*f* = 2, one-tailed paired *t* test. **g** Quantification of active Caspase-3 staining in Lsd1-iKO MG cells after treatment with vehicle or 5 µM 4-OHT. *p* Value = 0.0286, *t* = 4.003, d*f* = 2, one-tailed paired *t* test. **f**, **g** Staining and flow cytometric analysis for Ki67 and Caspase 3 were performed on fixed and permeabilized cells. Resulting values were normalized to vehicle control, and data shown represent the means of three biological replicates ± SEM

As shown above, our data suggest that Gfi1 interacts with Lsd1 and that this interaction may be important for tumor growth. To determine whether Lsd1 is required for growth of MG tumors, we crossed CAG-CreER^TM^ mice with Lsd1^fl/fl^ mice^21^ to obtain CAG-CreER^TM^; Lsd1^fl/fl^ mice (hereafter called Lsd1-inducible knockout, or Lsd1-iKO mice). We isolated neural progenitors from Lsd1-iKO pups and transduced them with *Myc* and *Gfi1* to generate Lsd1-iKO MG tumors. We then treated tumor cells with 4-OHT overnight to delete Lsd1. Assaying these cells by Western blotting confirmed that treatment with 4-OHT significantly reduced the amount of Lsd1 protein when compared to treatment with vehicle (DMSO) (Fig. 4a). When tumor cells were implanted into mice, those that had been treated with vehicle all gave rise to tumors (median survival = 19 days). In contrast, only 8/26 of mice that received 4-OHT-treated cells developed tumors, and they did so with increased latency (Fig. 4b, c). Importantly, the tumors that did arise from 4-OHT-treated cells expressed substantial amounts of Lsd1 protein, suggesting that these tumors arose from cells that had escaped Lsd1 deletion (Fig. 4d).

To understand the mechanisms by which loss of Lsd1 prevented tumor growth, we examined tumor cells for changes in proliferation and death after Lsd1 deletion. To measure proliferation, we treated Lsd1-iKO MG tumor cells with 1 or 5 μM 4-OHT for 48 h and performed 3-H Thymidine incorporation assays. Cells treated with both concentrations of 4-OHT showed markedly lower levels of incorporation compared to those treated with vehicle (DMSO), indicating that Lsd1 deletion impaired proliferation (Fig. 4e). Similarly, we observed a 63% decrease in the proportion of Ki67+ cells after treatment with 4-OHT (Fig. 4f, Supplementary Fig. 4a, b). We then looked for changes in cell death by staining cells for active Caspase 3, a marker for cells undergoing apoptosis. Tumor cells treated with 4-OHT exhibited 2.2-fold increase in active Caspase-3 expression, indicating that Lsd1 deletion also had a significant effect on tumor cell survival (Fig. 4g, Supplementary Fig. 4a, c). Staining with Annexin V and 7-AAD also showed a shift from live cells to dying and dead cells after 4-OHT treatment (Supplementary Fig. 4d, e).

To ensure that the effects of 4-OHT on tumor growth were caused by loss of Lsd1 and not by 4-OHT itself, we carried out parallel experiments using Lsd1^fl/fl^ MG tumor cells without the CreER^TM^ allele. Treatment of these tumor cells with 4-OHT did not activate CreER^TM^ and consequently did not delete Lsd1 (Supplementary Fig. 5a). Mice receiving vehicle-treated or 4-OHT-treated tumor cells developed tumors with 100% penetrance, similar latencies, and no difference in Lsd1 protein levels (Supplementary Fig. 5b–d). Thus, the effects of 4-OHT on tumor growth depend on deletion of Lsd1. Collectively, these findings demonstrate that Lsd1 is required for growth of MG tumors.

Since Lsd1 is expressed in many cancer types and is involved in a wide range of biological processes^22,23^, we sought to determine whether the inhibitory effect of Lsd1 deletion was specific to MG tumors. We therefore repeated the deletion experiments described above using cells from Lsd1-iKO MP tumors, which are driven by overexpression of *Myc* and *DNp53*. As shown in Supplementary Fig. 6a–c, loss of Lsd1 had a much more modest effect on MP tumors than it did on MG tumors: the majority of animals receiving 4-OHT-treated Lsd1-iKO MP tumor cells still developed tumors. Moreover, in contrast to the persistent expression of Lsd1 in Lsd1-iKO MG tumors, 10/12 of the tumors arising from Lsd1-iKO cells exhibited reduced expression of Lsd1 protein (Supplementary Fig. 6c). These results suggested that Lsd1 is dispensable for the growth of MP but not MG tumors.

### The p53 pathway remains functional in Gfi1-driven tumors

**Fig. 5:**
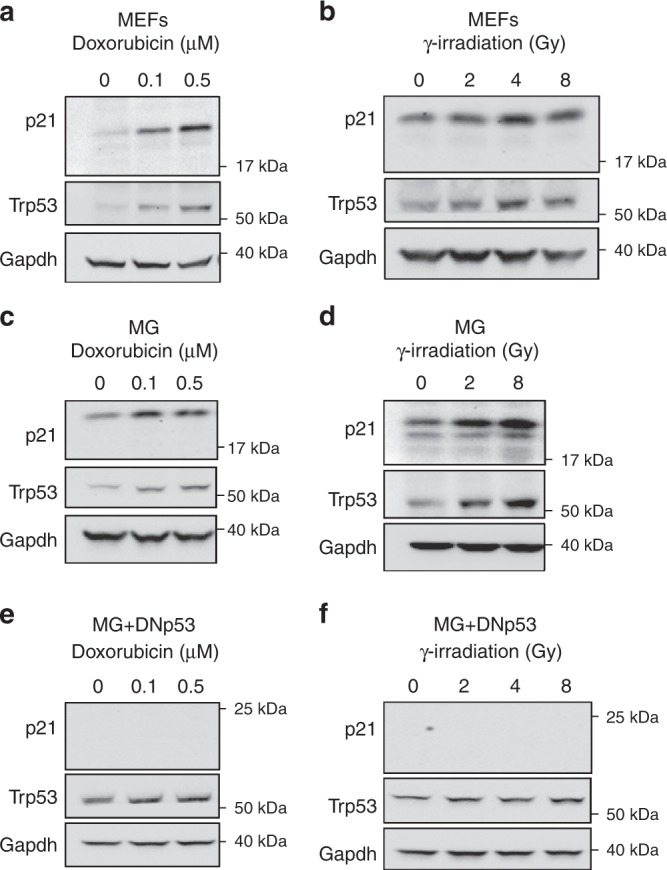
The p53 pathway remains functional in Gfi1-driven tumors. **a**, **b** Western blots for p53 and p21 protein in wild-type mouse embryonic fibroblasts (MEFs) after 4-h treatment with **a** 0, 0.1, or 0.5 μM doxorubicin or **b** 0, 2, 4, or 8 Gy of γ-irradiation. **c**, **d** Western blots for p53 and p21 protein in MG tumor cells after 4-h treatment with **c** doxorubicin or **d** γ-irradiation. **e**, **f** Western blots for p53 and p21 protein in MG tumor cells expressing DNp53 after 4-h treatment with **e** doxorubicin or **f** γ-irradiation

The studies described above show that Gfi1 depends on interactions with Lsd1 to promote tumor growth, but the signaling pathways and target genes affected by Gfi1 and its cofactors remain unknown. In the hematopoietic system, several groups have reported that Gfi1 can repress genes in the p53 pathway^18,24,25^. Furthermore, two previously established models of MB combine *Myc* overexpression with p53 loss of function^4,5^. Based on these observations, we wondered whether Gfi1 might contribute to MB growth by suppressing the p53 pathway. To assess the activity of the pathway, we treated cells with doxorubicin or with γ-irradiation to elicit p53-dependent DNA damage responses. After 4 h, samples were analyzed by Western blotting for p53 and its downstream effector p21. Mouse embryonic fibroblasts (MEFs), which express wild-type p53, showed increased expression of p53 and its target p21 in response to doxorubicin and γ-irradiation (Fig. 5a, b). Likewise, doxorubicin and γ-irradiation caused dose dependent increases in p53 and p21 in MG tumors (Fig. 5c, d). To confirm that p53 inactivation can desensitize MB cells to these treatments, we overexpressed DNp53 in MG tumors and then analyzed their response to doxorubicin and γ-irradiation. While DNp53-expressing MG tumor cells exhibited accumulation of p53 protein (because DNp53 prevents upregulation of the Mdm2 ubiquitin ligase that would otherwise promote p53 degradation), they no longer showed induction of p21 in response to DNA damage (Fig. 5e, f).

Given that both Gfi1 and Lsd1 are necessary for MG tumors, it is also notable that others have demonstrated Lsd1 demethylation of p53 protein, which destabilizes it and prevents its association with coactivators^25,26^. We thus considered the possibility that MG tumors might have increased Lsd1 expression levels that could lead to reduced p53 activity. However, Lsd1 levels in MG tumors were not higher than those in NSCs, from which MG tumors are derived (Supplementary Fig. 7). Together, these results suggest that MG tumors have normal p53 function and that it is unlikely that the critical role of Gfi1 in MG tumorigenesis is to repress p53.

### Neuronal differentiation genes are decreased in MG tumors

**Fig. 6:**
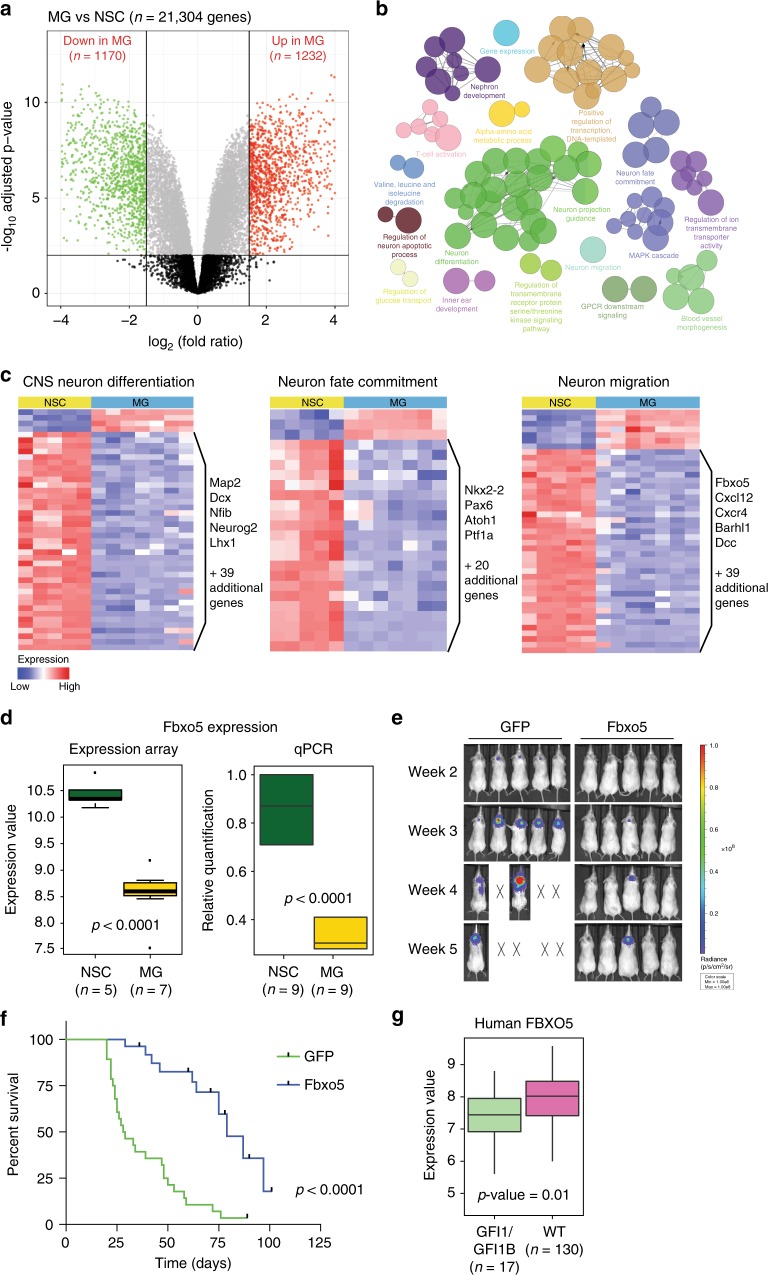
Neuronal commitment and differentiation pathways are downregulated in MG tumors. **a** Volcano plot of of 21,304 genes whose expression was compared in MG tumors (*n* = 7) and NSCs (*n* = 5). Red indicates genes that were significantly upregulated in MG (log_2_FC > 1.5 and FDR-corrected *p* value < 10^−2^) and green indicates those that were significantly downregulated in MG (log_2_FC < −1.5 and FDR-corrected *p* value < 10^−2^). **b** Pathway analysis of differentially expressed genes. Each node represents a GO term. Node size reflects the enrichment significance of the term. Edges represent association strength between the terms calculated using chance corrected kappa statistics (a standard kappa score level threshold was used). **c** Heat maps comparing expression of genes involved in central nervous system neuron differentiation (left), neuron fate commitment (center), and neuron migration (right) in MG tumor cells and NSCs. **d** Fbxo5 expression from microarray analysis comparing NSCs to MG tumor cells (left) and qPCR validation of Fbxo5 mRNA expression in MG tumor samples compared to NSC samples (right). *p* values < 0.0001 were determined by one-sided Welch Two Sample *t* test. Whiskers indicate minimum and maximum expression values, bottom of boxes indicate first quartiles, midline indicates median expression values, and top of boxes indicate third quartiles. **e** Bioluminescence imaging of mice transplanted with cells infected with empty vector control (GFP) or Fbxo5. X’s denote animals euthanized before they could be imaged. **f** Survival curves comparing mice transplanted with cells infected with empty vector (GFP) or Fbxo5 (GFP *n* = 28, Fbxo5 *n* = 27). *p* Value < 0.0001 was determined by Log-rank (Mantel–Cox) test. **g** FBXO5 expression in human Group 3/Group 4 MB samples with or without GFI1/GFI1B activation (GFI1/GFI1B *n* = 17, WT *n* = 130). *p* Value was determined by one-sided Welch Two Sample *t* test. Whiskers indicate minimum and maximum expression values, bottom of boxes indicate first quartiles, midline indicates median expression values, and top of boxes indicate third quartiles

To identify other potential mechanisms by which Gfi1/Lsd1 promote tumor formation, we analyzed transcriptional profiles obtained from MG tumors (*n* = 7) and NSCs (*n* = 5). Our analysis identified a total of 2402 differentially expressed genes, of which 1170 were downregulated ((FDR)-adjusted *p* value < 10^−2^, log_2_FC < −1.5) and 1232 were upregulated ((FDR)-adjusted *p* value < 10^−2^, log_2_FC > 1.5) (Fig. 6a). Pathway analysis of differentially expressed genes revealed an enrichment of genes associated with neuronal fate commitment and differentiation, neuron migration, neuron projection guidance, and neuron apoptotic processes (Fig. 6b); regulators of these processes were expressed at significantly lower levels in MG tumors than in NSCs (Fig. 6c). Consistent with this, many of the most downregulated genes in MG tumors have functions in neuronal differentiation and migration: *Ptf1a*^27^, *Sox11*^28^, *Zic3*^29^, *Dcc*^30^, *Neurog2*^31^, *Zic1*^32^, *Barhl1*^33^, *Neurod1*^34^, *Nfib*^35^, and *Fbxo5*^36^ (Supplementary Data 1). These findings suggest that repression of neuronal commitment and differentiation may play a key role in the transformation of normal NSCs into MG tumors.

As an additional approach to elucidate the targets of Gfi1, we performed chromatin immunoprecipitation and sequencing (ChIP-seq) in MG tumors and identified 10,840 significant peak regions bound by Gfi1 (Supplementary Data 2). Since we have demonstrated that Lsd1 is an essential cofactor of Gfi1 in MG tumors, we predicted that Gfi1/Lsd1 might co-occupy similar genomic regions and therefore carried out ChIP-seq using antibodies specific for Lsd1 as well. We identified 12,083 peaks where Lsd1 was bound and confirmed a high concordance between Lsd1 and Gfi1 peaks (Supplementary Fig. 8a, Supplementary Data 2): 9594 peaks were common to both Gfi1 and Lsd1 datasets, representing ~89% of all Gfi1 peaks and ~79% of all Lsd1 peaks (Supplementary Fig. 8b). The sizable overlap between Gfi1 and Lsd1 binding in the genome further substantiates that these proteins interact to co-regulate common downstream target genes and pathways.

To evaluate the functional relevance of predicted Gfi1 target genes, we tested a subset of the genes that were bound by Gfi1 and differentially expressed in MG tumors compared to NSCs. We focused specifically on genes that have been reported to regulate differentiation, which were downregulated in MG tumors (Fig. 6d, Supplementary Fig. 9a, c, e, g). We hypothesized that genes whose downregulation was critical for tumorigenesis might inhibit tumor growth if they were re-expressed in tumor cells. Therefore, we used retroviruses to overexpress these genes in tumor cells and examined the effects on tumor growth in vivo. While the majority of genes we tested did not affect tumor growth or latency (Supplementary Fig. 9b, d, f, h), overexpression of *Fbxo5* caused a significant delay in tumor formation (Fig. 6e, f). Notably, analysis of human MB showed that FBXO5 levels were lower in GFI1/1B-activated Group 3/Group 4 tumors than in tumors without GFI1/1B-activation (Fig. 6g). Together these findings suggest that *Fbxo5* may be a downstream effector of *Gfi1* in MB.

### Pharmacological inhibition of Lsd1 to treat Gfi1-driven MB

**Fig. 7:**
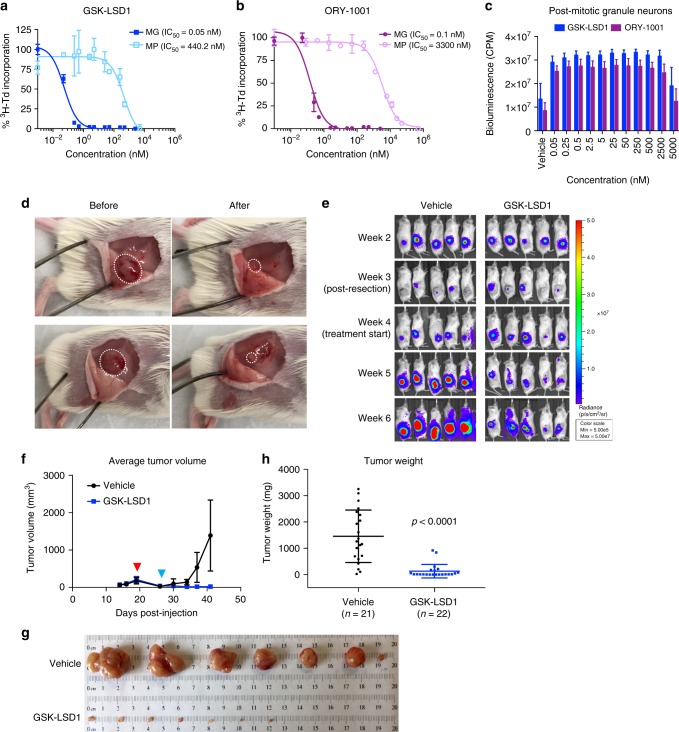
Pharmacological inhibitors of Lsd1 inhibit growth of Gfi1-driven MB. **a**, **b** In vitro proliferation assays for MG and MP tumor cells treated with **a** GSK-LSD1 or **b** ORY-1001. Proliferation was measured via ^3^H-thymidine incorporation at 48hrs. **c** Cell viability of post-mitotic granule neurons treated with GSK-LSD1 (blue) or ORY-1001 (purple). Viability was measured via CellTiter-Glo Luminescent Assay at 48 h. Data shown in **a**–**c** are from representative experiments and are plotted as the means of technical triplicate samples ± SEM. All experiments were repeated in at least three biological replicates. **d**–**h** Mice implanted with subcutaneous MG tumors underwent surgical resection of tumors and were subsequently treated with vehicle (saline) or 10 mg/kg GSK-LSD1 in cycles of four days on and three days off (vehicle *n* = 21, GSK-LSD1 *n* = 22). **d** Representative images of two tumors before (left) and after (right) surgical resection. Tumors are outlined by white dotted lines. Tumor growth was monitored weekly by **e** bioluminescent imaging and **f** caliper measurements. Red arrowhead indicates time of surgical resection. Blue arrowhead indicates start of drug treatment. Data shown are from a representative experiment and are plotted as the means ± SEM. When tumors reached the maximum allowed diameter, mice were sacrificed and resulting tumors were **g** collected and **h** weighed. Compared to resection alone, resection and GSK-LSD1 treatment significantly reduced tumor burden in mice. *p* < 0.0001, *t* = 6.042, d*f* = 41, one-tailed unpaired *t* test. Data shown in **h** are from three replicate experiments and plotted as the means ± SD

Based on our finding that the interaction between Gfi1 and Lsd1 is crucial for MG tumor growth, we sought to determine whether small molecule inhibitors of Lsd1 could serve as therapeutic agents for these tumors. We performed thymidine incorporation assays on cells treated with two different Lsd1 inhibitors: GSK-LSD1 and ORY-1001. Both compounds potently inhibited proliferation of MG tumor cells in vitro, with IC_50_s ranging from 0.05 to 0.1 nM (Fig. 7a, b). In contrast, the effect on MB tumor models not driven by Gfi1 was much less pronounced. As shown in Fig. 7a, b, the proliferation of MP tumor cells was inhibited only with much higher concentrations of these compounds (IC_50_ = 440–3300 nM). Cells derived from Glt1-tTA:TRE-MYCN/Luc (GTML) and Math1-Cre;Ptch1^fl/fl^ Luciferase (MPL) tumors, models of Group 3 and SHH MB respectively, were also relatively insensitive to Lsd1 inhibitors (Supplementary Fig. 10a, b). Finally, we tested the effects of GSK-LSD1 and ORY-1001 on normal granule neurons, and did not see adverse effects on viability (Fig. 7c). These data suggest that pharmacological inhibition of Lsd1 potently and selectively inhibits proliferation of Gfi1-activated tumor cells.

To test whether Lsd1 inhibition also impedes tumor growth in vivo, we implanted MG tumors into the flanks of NSG mice and treated them with vehicle (4% DMSO in saline) or 10 mg/kg GSK-LSD1. Tumor growth was monitored weekly by bioluminescent imaging and caliper measurements (Supplementary Fig. 11a, b). When tumors reached 2 cm in diameter, the experiment was terminated, and tumors were collected and weighed (Supplementary Fig. 11c, d). As shown in Supplementary Fig. 11a-d, treatment with GSK-LSD1 significantly slowed tumor growth and decreased the size of MG tumors in vivo. We also tested the effects of Lsd1 inhibitors on mice bearing intracranial MG tumors, but saw no effects on tumor growth or survival (Supplementary Fig. 12). The fact that we observed inhibition of MG tumors in the flank but not in the brain suggests that these compounds do not accumulate to sufficient levels within brain tumors to exert a therapeutic effect.

The experiments above tested the effects of Lsd1 inhibitors alone. Because standard therapy for MB includes surgery and radiation, we asked whether Lsd1 inhibitors could be combined with these modalities. To test the combination with surgical resection, we implanted MG tumors into the flanks of NSG mice, and when tumors reached a volume of 200–300 mm^3^, we performed surgery to remove the bulk of the tumor (Fig. 7d). After 1 week of recovery, mice were randomized into treatment groups based on tumor size, and treated with vehicle (saline) or 10 mg/kg GSK-LSD1. Tumor growth was monitored via bioluminescent imaging and caliper measurements, and endpoint tumors were collected and weighed. As shown in Fig. 7e–h, GSK-LSD1 was highly effective at suppressing tumor growth following surgical resection. GSK-LSD1 also potently inhibited tumor growth after ionizing radiation (Supplementary Fig. 11e–h). These results strongly support the notion that targeting Lsd1 with small molecule inhibitors could be an effective strategy for treating patients with Gfi1-driven MB.

## Discussion

Our previous studies demonstrated that GFI1 and GFI1B can cooperate with MYC to drive tumorigenesis in a subset of Group 3 and Group 4 MBs^6^, but the means by which they promote MB growth are not well understood. In the present study, we not only demonstrate the importance of Gfi1 expression in tumor maintenance, but also show that its tumorigenic activity depends on its ability to recruit other proteins through a functional SNAG domain. In particular, we identify the epigenetic modifier Lsd1 as a critical partner of Gfi1, and we demonstrate that these proteins promote oncogenic transformation in part by inhibiting neuronal differentiation. Furthermore, we show that Lsd1 inhibitors selectively inhibit growth of MG tumors, highlighting Lsd1 inhibition as a potential strategy for treatment of patients with Gfi1-driven MB.

Identifying genes required for tumor maintenance is a major challenge in cancer biology. The genetic alterations that drive the early stages of tumorigenesis are not always maintained at later stages, and tumors often accumulate additional mutations that render initiating oncogenes dispensable for continued tumor growth^37,38^. When this occurs, these genes may not be useful as targets for therapy. Using a conditional genetic deletion approach, we show that Gfi1 is not only required for tumor initiation but is also crucial for maintaining growth of established tumors. This implies that targeting GFI1 may benefit patients whose tumors overexpress this oncogene. Although direct inhibition of GFI1 is not currently possible, our studies suggest that targeting its cofactors may be a viable approach to therapy.

Our finding that in MG tumors Gfi1 exists in a complex that includes CoREST and Lsd1 is consistent with interactions described in other systems^11–14^. The importance of Lsd1 as a mediator of tumorigenesis is supported by our findings that mutation of the SNAG domain, which is critical for Lsd1 recruitment^13,14,19^, abolishes the tumorigenic potential of Gfi1, and that Cre-mediated deletion of Lsd1 prevents propagation of MG tumors. Consistent with these results, our ChIP-seq experiments revealed that 80% of the sites bound by Gfi1 were also bound by Lsd1. Recruitment of Lsd1 to target genes can result in either transcriptional repression or transcriptional activation: by demethylating histone 3 lysine 4 (H3K4), Lsd1 removes a mark associated with activation, leading to repression of target genes. Conversely, Lsd1-mediated demethylation of H3K9 eliminates a repressive mark, resulting in transcriptional activation. In line with this, our comparison of gene expression and ChIP-seq analyses revealed activation and repression of subsets of genes bound by Gfi1.

Our previous studies demonstrated that inactivation of p53 can cooperate with Myc to promote MB formation^4,5^. The fact that Gfi1 can also cooperate with Myc to drive tumor growth^6^, along with reports that Gfi1 can antagonize p53 and its targets^18,24,25^, led us to speculate that Gfi1 might promote MB formation by repressing p53 function. However, we observed normal induction of p53 and p21 following exposure to DNA damaging agents, suggesting that p53 is still capable of being activated in MG tumors. Together these data suggest that that the ability of Gfi1 to cooperate with Myc does not depend on inactivation of p53. However, we cannot rule out the possibility that Gfi1 acts in part by regulating pathways that are also regulated by p53.

To gain insight into the mechanisms by which Gfi1 promotes tumor formation we performed expression profiling on MG tumor cells. Among the most prominent pathways altered in these tumors were those related to neuronal differentiation. We found that many of the associated genes were downregulated in MG tumors compared to NSCs, suggesting that one important aspect of transformation is repression of neuronal differentiation. This is consistent with our previous observation that MG tumors are poorly differentiated and do not express markers of mature neurons^6^. The importance of maintaining a proper balance between self-renewal and differentiation has been observed in brain tumors as well as many other types of cancer^39–42^. Since NSCs are the presumed cell of origin in MG tumors, our study suggests that transformation of NSCs involves not only increased proliferation (driven by MYC) but also failure to undergo normal differentiation (mediated at least in part by Gfi1).

To identify target genes whose repression is important for MG tumor growth, we tested whether overexpression of these genes could impair tumor formation. While most of the genes we tested had no effect on tumor growth, Fbxo5 significantly reduced tumorigenicity. Previous studies have suggested that Fbxo5 (also known as Emi1) is a mitotic regulator that acts to inhibit the anaphase promoting complex (APC), allowing Cyclin B1 accumulation and cell cycle progression from G1 to S phase^43,44^. Consistent with this, overexpression of Fbxo5 has been suggested to promote cell proliferation and tumorigenesis^45–47^. However, Fbxo5 has also been shown to induce mitotic arrest and apoptosis^45^, and to promote neuronal differentiation. For example, inhibition of APC/Cdh1 by Fbxo5 in cerebellar granule neurons causes increased axonal growth^36^, and FBXO5 overexpression in mesenchymal stem cells promotes migration and differentiation^48^. Our observations that Fbxo5 is repressed in MG tumors and can inhibit growth of these tumors following transplantation support the possibility that Fbxo5 inhibition might play an important role in mediating the transforming effects of Gfi1.

Because of the relatively poor prognosis and paucity of treatment options available for Group 3 MB, there is a critical need to identify drugs that are effective against these tumors. Two recent high-throughput drug screens have identified potential candidates for combination therapies^49–51^, but both screens were carried out using MB models driven by Myc and loss/inactivation of p53, so they do not represent the subset of tumors with Gfi1/1b activation. Therefore, our observation that Lsd1 inhibitors can block growth of MG tumor cells alone and in combination with surgery and radiotherapy may have significant implications for patients with GFI1-driven MB. Notably, we observed a stark difference in efficacy when using Lsd1 inhibitors to treat subcutaneous vs. intracranial MG tumors. The lack of effect on intracranial tumors suggests that the Lsd1 inhibitors we tested are limited in their ability to penetrate brain tumors. Further studies will be necessary to determine whether other Lsd1 inhibitors, or alternative modes of delivery, may increase the utility of these drugs for intracranial tumors. If these approaches are successful, Lsd1 inhibition could represent a promising targeted therapy for patients with Gfi1/1b-activated MB.

## Methods

### Virus production

Totally, 8 × 10^6^ HEK 293T/17 cells (ATCC CRL­11268) were plated in T150 cell culture flasks 1 day prior to Calcium Phosphate transfection. On the day of transfection, media was exchanged 0.5–2 h before transfection. Sterile distilled H_2_O, plasmids (VSVG, Gag/Pol, and vector of interest), and 2 M CaCl_2_ were prepared in one tube. 2× HEPES (280 mM NaCl, 1.5 mM Na_2_HPO_4_, 50 mM HEPES free acid; pH 7.0–7.1) was added dropwise to the tube containing H_2_O/plasmids/CaCl_2_ and incubated for 1 min at room temperature. Mixture was added onto cells and swirled very gently to mix. After incubation at 37 °C for at least 5 h, media was exchanged and cells were returned to the incubator. Viral supernatant was harvested at 24, 48, and 72 h after transfection and stored at 4 °C until concentration step. To generate concentrated virus, supernatants were filtered through 0.45 µM filters and then centrifuged at 25,000 RPM, 4 °C for 2 h using the SW32 rotor in an Optima L-80 XP ultracentrifuge. After centrifugation, supernatant was removed and the viral pellets were resuspended in ~500 µl media, aliquoted, frozen on dry ice, and stored at −80 °C until further use. Virus titration was carried out by infecting 293T cells with serial dilutions of virus and analyzing reporter expression by flow cytometry (GFP or dsRed) or by bioluminescence plate reader (luciferase).

### Retroviral constructs

Retroviruses used for generation of MG tumors include: MSCV-Myc^T58A^-IRES-GFP, MSCV-Myc^T58A^-IRES-Luciferase, MSCV-Myc^T58A^-IRES-CD2, MSCV-Gfi1-IRES-GFP, MSCV-Gfi1-IRES-Luc, and MSCV-loxp-Gfi1-loxp-IRES-GFP. Cloning of the Myc^T58A^ and Gfi1 constructs have been described previously^4,6^. MSCV-loxp-Gfi1-loxp-IRES-GFP was cloned by ploymerase chain reaction (PCR) amplification of *Gfi1* using primers with loxp sites and EcoRI/XhoI sites added to the ends.

Retroviruses used for testing the Gfi1 domain functions include pSF91-empty vector (control), pSF91-Gfi1-dsRed (wild-type), pSF91-Gfi1^P2A^-dsRed (SNAG mutant).

Retroviruses used for testing functional relevance of candidate Gfi1 target genes include: MSCV-Fbxo5-IRES-GFP, MSCV-Bmpr1a-IRES-GFP, MSCV-Lrig3-IRES-GFP, MSCV-Nfia-IRES-GFP, and MSCV-Smad4-IRES-GFP. Genes were cloned by PCR amplification of cDNAs obtained from the Mammalian Gene Collection (Dharmacon) and ligation into the MSCV-IRES-GFP vector.

### Co-immunoprecipitations

MG tumor cell pellets (at least 3 × 10^6^ cells) or Gfi1-infected NIH-3T3 cells (ATCC CRL-1658) were resuspended in lysis buffer (150 mM NaCl, 20 mM Tris, 1% Triton X-100) with protease inhibitors (Roche, cat #1836153). Lysates were precleared for 30 min with Protein G agarose beads (Millipore #16-201D, Cell Signaling #37478). Totally, 10% of the sample was saved as input, and the rest was split in half for immunoprecipitation with experimental and control antibodies. Samples were incubated with antibodies specific for Gfi1 (1 µg, Santa Cruz sc-8558), Lsd1 (1 µg, Abcam ab17721) or CoREST (1 µg, Millipore cat #07-455), or with isotype control antibodies (1 µg, Santa Cruz sc-2028) for 1 h. Protein G beads were added to the samples and washed three times before preparing for Western blot by adding 4× sodium dodecyl sulfate (SDS) sample buffer and boiling.

### Western blotting

Immunoprecipitated samples prepared as described above were run on 10% SDS-PAGE gels, transferred to nitrocellulose membranes, blocked with 5% nonfat milk (Apex) in tris-buffered saline with 0.1% Tween-20 (TBST), and stained overnight with anti-Gfi1 (1:500, Abcam ab21061), anti-Lsd1 (1:800, Abcam ab17721), anti-CoREST (1:2000, Millipore cat #07-455), anti-HDAC1 (1:1000, Cell Signaling #5356), or anti-HDAC2 (1:1000, Cell Signaling #5113) antibodies. Membranes were incubated for 1 h with anti-rabbit HRP-conjugated secondary antibody (1:1000, Cell Signaling #7074) followed by visualization with Clarity Western ECL (Bio-Rad, cat #170-5060) on the ChemiDoc MP Imaging System (Bio-Rad).

All other samples for Western blotting were lysed in 1× RIPA buffer (Millipore, cat #20-188) and quantified using the Pierce BCA protein assay kit (ThermoFisher cat #23225). Protein separation, transfer, and blocking were as described above. 10% SDS-PAGE was used for detection of Gfi1, Lsd1, and CoREST. Totally, 12% SDS-PAGE was used for detection of p53 and its targets. In addition to anti-Gfi1, anti-Lsd1, and anti-CoREST antibodies mentioned above, other primary antibodies used include anti-Gapdh (1:1000, Cell Signaling #2118), anti beta-Actin (1:1000, Cell Signaling #4967), anti-p21 (1:200, Santa Cruz sc-6246), and anti-p53 (1:1000, Cell Signaling #2524). Membranes were incubated with anti-rabbit HRP-conjugated secondary (1:1000, Cell Signaling #7074) or anti-mouse HRP-conjugated secondary (1:1000, Cell Signaling #7076). Protein visualization was as described above.

### Induction of p53 DNA damage response

MEFs were isolated from C57BL/6 embryos at embryonic day 14.5. MEFs were plated at 1 × 10^6^ cells/well and MG tumor cells were plated 3–5 × 10^6^ cells/well in 6-well plates. Cells were treated with vehicle (DMSO), 0.1 or 0.5 µM doxorubicin (Cayman Chemical cat #15007), or they were irradiated with 0, 1, 2, 4, or 8 Gy using a low-dose cesium 137 irradiator at the Sanford Burnham Prebys Animal Facility. Samples were collected 4 hrs after treatment or irradiation for analysis of p53 and p21 protein levels by Western blot.

DNp53-expressing MG tumors were generated by isolating MG tumor cells, infecting with MSCV-DNp53-IRES-GFP viruses, sorting for infected cells and retransplanting the cells into new NSG mice. These tumors were later harvested and their cells were treated with doxorubicin or irradiated as described above.

### Mice

Mouse strains used in these studies include: C57BL/6, Lsd1^fl/fl^ ^21^ (a gift from Michael G. Rosenfeld, UCSD), Tg(CAG-cre/Esr1*)5Amc/J (CAG-CreER^TM^) (JAX Stock #004453)^10^, and NOD-*scid* IL2Rgamma^null^ (NSG). Lsd1^fl/f^ mice were bred with CAG-CreER^TM^ mice to produce mice with a CAG-CreER^TM^; Lsd1^fl/fl^ genotype. C57BL/6, Lsd1^fl/fl^, CAG-CreER^TM^, and CAG-CreER^TM^ Lsd1^fl/fl^ pups were all used as sources of Prominin + neural progenitor cells^9^. NSG mice were used as transplantation hosts.

Mice were bred and maintained at the Sanford Burnham Prebys (SBP) Medical Discovery Institute and Sanford Consortium Animal Facilities. Experiments were performed in accordance with national regulations using procedures approved by the Institutional Animal Care and Use Committees at SBP Discovery and the University of California San Diego. No a priori calculations related to sample size were performed.

### Orthotopic transplantation and tumor formation

Primary MG and MP tumors were generated by isolating Prominin1+ (CD133+) neural stem cells from mice, infecting them with retroviruses encoding *Myc*^*T58A*^ and either *Gfi1* or *DNp53*, and transplanting the cells into host NSG mice. The isolation of neural stem cells has been described previously;^9^ in general, cerebella from postnatal day 4–6 mice were dissected and enzymatically dissociated into single cell suspension. Cells were subjected to Percoll fractionation (GE Healthcare Life Sciences 17-0891-02), stained with anti-mouse CD133 PE antibody (1:200, eBiosience 12-4301-82), and sorted for the Prominin + population (approximately 3–4% of cells). The isolated cells were infected with the appropriate viruses and the next day, 5 × 10^4^ cells were resuspended in Neurocult NSC Basal medium (Stem Cell Technologies, cat #05700) with Neurocult NSC Proliferation Supplement (Stem Cell Technologies, cat #05701) and injected into the cerebella of NSG mice (age 6–8 weeks) using a stereotactic frame equipped with mouse adaptor (David Kopf Instruments). Animals were monitored weekly, and at the time of sacrifice, brains were removed for tumor dissection and dissociation. The method for tumor dissociation was identical to that described above for the isolation of NSCs from the cerebellum.

### Genetic deletion of Gfi1 or Lsd1 from MG tumors

To initiate deletion of Gfi1 from CAG-CreER^TM^ MG^flox^ or Lsd1 from CAG-CreER^TM^ Lsd1^fl/fl^ MG tumors, tumors were dissociated and cultured overnight in DMEM (with l-glut, 4.5 g/L glucose and sodium pyruvate), 10% FBS, and 1× Pen/Strep supplemented with either vehicle (DMSO) or 5 µM 4-hydroxytamoxifen (4-OHT, Sigma cat #H7904). The following day, the cells were washed, counted, and allocated for (1) orthotopic retransplant into NSGs to assess growth changes in vivo and (2) replating in fresh media without vehicle or 4-OHT for deletion analysis by Western blot after an additional 72 h. To determine changes in proliferation and apoptosis after Lsd1 deletion, CAG-CreER^TM^ Lsd1^fl/fl^ MG tumors cells treated with DMSO or 4-OHT were also analyzed after 40–48 h using Thymidine incorporation assays (described below) and flow cytometric analysis for Ki67 (BD Biosciences cat #561126), active Caspase 3 (BD Biosciences cat #560626), and Annexin V (BD Biosciences cat #550474) staining.

### Gene array analysis

Microarray gene expression data for 7 MG and 5 NSC samples were obtained using the GeneChip Mouse Genome 2.0 Array from Affymetrix. The raw data were preprocessed using the RMA algorithm that includes background correction, normalization, and calculation of expression steps. The resulting expression values were in log base 2 scale. Differential gene expression analysis was performed in the R statistical environment: *p* values were calculated using a linear model fit from the “limma” package and the BH method was used for multiple testing correction. Differentially expressed genes were defined using a cut-off of absolute value of the log fold change between average expression values in MG and NSC cases of 1.5 and of corrected *p* values of 10^−2^ (*n* = 2402 genes). Pathway analysis of the differentially expressed genes was performed using ClueGO plug-in for Cytoscape.

### ChIP-sequencing and target identification

Chromatin extraction, immunoprecipitation, and library preparation for ChIP-seq were performed at Active Motif (Carlsbad, CA). For Gfi1 ChIP, 30 ug of chromatin was subjected to immunoprecipitation with 4 µg of rabbit polyclonal, anti-Gfi1 antibody (GNE-CJS-2; kindly provided by H. Leighton Grimes). For Lsd1 ChIP, 30 µg of chromatin was subjected to immunoprecipitation with 4 µg of rabbit polyclonal, anti-Lsd1 antibody (Abcam #ab17721). Sequencing of libraries was performed at the German Cancer Research Center (DKFZ; Heidelberg, Germany). Sequence quality was assessed by FastQC (https://www.bioinformatics.babraham.ac.uk/projects/fastqc/).

Sequence reads from ChIP-Seq were aligned to the mouse reference genome (mm10, chromosomes 1-19, X, Y, and M) using the “Burrow–Wheeler Transformation” based aligner BWA (version 0.6.2, arguments −q20)^52^. BAM files were merged using Picard (http://picard.sourceforge.net) and duplicate reads were removed.

Peak calling for Gfi1 and Lsd1 in MG tumors was done using MACS with default parameters and using the appropriate input chromatin controls, used in the preparation of the respective factor ChIPs. To identify the high-confidence peak set for each factor, initially the peaks with the upper 20th percentile enrichments were identified separately for the two replicates of the respective factor. Afterwards, resulting peaks were overlapped (at least 50% overlap) and the overlapping peaks were referred as the high confidence peak set of the respective factor. Gfi1 target genes were determined by identifying the genes with transcriptional start sites closest to the respective peaks. Gene annotations were based on gencode vM9 (http://www.gencodegenes.org/mouse_releases/0.html).

### Comparison of Gfi1 and Lsd1 signal

We quantified the coverage of Gfi1 and Lsd1 at each base pair in the region surrounding ±3 kb midpoint Gfi1 high confidence peaks. Read coverage was averaged in 200-bp windows along the regions and the values were scaled to arrange between 0 and 1. After ordering the values according to descending Gfi1 signal intensity, resulting values were represented as heat maps.

### Quantitative PCR (real-time PCR)

To validate expression of Gfi1 and Lsd1 target genes, mRNA was isolated from cells using an RNeasy Plus Mini kit (Qiagen). RNA was reverse transcribed to cDNA using iScript Reverse Transcription Supermix (Bio-Rad, cat #1708841), and duplicate no RT reactions were also prepared to confirm absence of genomic contamination. Then qPCR reactions were performed in triplicate using iQ SYBR Supermix (Bio-Rad, cat #1708882) on the Bio-Rad C1000 Thermocycler and CFX96/CFX384 systems. Relative gene expression was calculated using the ∆∆CT method and normalized to Actin. 95% confidence intervals for each sample were calculated using the sum of squares method.

### Thymidine incorporation assay

For Lsd1 deletion experiments, CAG-CreER^TM^ Lsd1^fl/fl^ MG tumor cells were cultured in 96-well plates at 5 × 10^4^ cells/well and treated with vehicle (DMSO), 1 or 5 µM 4-OHT for 48 h before being pulsed with [methyl-^3^H] thymidine (Perkin Elmer, NET027A250UC). For drug treatment experiments, MG tumor cells were cultured in 96-well plates at 5 × 10^4^ cells/well, while Math1-Cre Ptch1^fl/fl^ Luciferase (MPL) tumor cells were cultured in 96-well plates at 2 × 10^5^ cells/well. Cells were treated with different concentrations of GSK-LSD1 (Cayman Chemical, cat #16439), ORY-1001 (Roche), or RN-1 (EMD Millipore, cat #489479) for 48 h before being pulsed. After 16–18 h, cells were frozen at −80 °C to stop incorporation and later harvested using a Mach IIIM manual harvester 96 (Tomtec). Incorporated radioactivity was quantitated using a Wallac MicroBeta TriLux microplate scintillation counter (Perkin Elmer). IC50s were determined using nonlinear regression analysis in GraphPad Prism.

### Cell viability assay

Granule neuron progenitors were isolated from 7 day-old C57BL/6 pups and cultured at a density of 2 × 10^5^ cells/well in 96-well plates in differentiation media (neurobasal + NS-21 supplement containing 25 mM glucose and 25 mM potassium chloride) for 5 days. Glt1-tTA:RE-*MYCN*/Luciferase (GTML) tumor cells were cultured in 96-well plates at 5 × 10^4^ cells/well. Cells were treated with different concentrations of GSK-LSD1 (Cayman Chemical, cat #16439) or ORY-1001 (Roche) for 48 h. To assess viability, we used the Cell TiterGlo luminescent assay (Promega, cat #G7570) and added the reagent 1:1 to cultured cells. Bioluminescence was read using the EnVision plate reader (Perkin Elmer).

### Flank tumor implantation and in vivo drug treatment

MG tumors were dissociated into single cell suspensions and mixed 1:1 with growth factor-reduced matrigel (BD Biosciences, cat # 354230). A total of 50,000 tumor cells suspended in 100 µl were injected into the flanks of NSG mice. One week after injection, tumor size was measured by in vivo bioluminescent imaging using the Xenogen Spectrum imaging system. Mice were randomized into two groups based on tumor size. Mice received i.p. injections of either vehicle (4% DMSO in saline) or 10 mg/kg of GSK-LSD1 (Cayman Chemical, cat #16439); each week, treatments were administered for 4 consecutive days followed by a 3-day holiday. Tumor growth was monitored weekly by both bioluminescent imaging and caliper measurements. When tumors reached 2 cm in diameter, experiments were terminated and mice in both cohorts were sacrificed. Tumors were collected, weighed, and photographed.

For experiments combining surgical resection and drug treatment, tumors were implanted as described above and monitored by bioluminescent imaging and caliper measurements. Once tumors reached a volume of 200–300 mm^3^, surgery was performed to remove the tumor bulk. Mice were given preop local anesthetic Marcaine (8 mg/kg, s.c.), isofluorane (4% in 2 L/min of O_2_) during the procedure, and postop analgesic Rimadyl (4 mg/kg, s.c.). Incisions were made in the skin surrounding the tumor, and the majority of tumor was removed. Vetbond tissue adhesive (Fisher Scientific cat #50822189) was used to close the incisions. After 1 week of recovery, mice were randomized based on tumor size, and treatment with vehicle (saline) or GSK-LSD1 began as described above. Tumor monitoring and collection criteria remained the same.

For experiments combining radiation therapy and drug treatment, tumors were implanted as described above and monitored by bioluminescent imaging and caliper measurements. Once tumors reached an average bioluminescent signal of ~5 × 10^8^ photons per second, mice were irradiated with 5 doses of 3 Gy given every other day (excluding weekends). Mice were sedated prior to irradiation with a cocktail of ketamine (100 mg/kg) and xylazine (10 mg/kg). Lead shielding was used to minimize radiation exposure to areas without tumor. After the course of irradiation was finished, mice were randomized into two groups based on tumor size, and treatment with vehicle (saline) or GSK-LSD1 began as described above. Tumor monitoring and collection criteria remained the same.

### Reporting summary

Further information on experimental design is available in the Nature Research Reporting Summary linked to this article.

## Supplementary information

Supplementary Information

Description of Additional Supplementary Files

Supplementary Data 1

Supplementary Data 2

Reporting Summary

## Data Availability

All genomics data that support the findings of this study have been deposited in the National Center for Biotechnology Information Gene Expression Omnibus (GEO) and are accessible through the GEO Series accession numbers GSE123409 [https://www.ncbi.nlm.nih.gov/geo/query/acc.cgi?acc=GSE123409] (expression microarray), GSE123870 [https://www.ncbi.nlm.nih.gov/geo/query/acc.cgi?acc=GSE123870] (ChIP-seq), and GSE123871 [https://www.ncbi.nlm.nih.gov/geo/query/acc.cgi?acc=GSE123871] (SuperSeries). All other relevant data are available from the corresponding author on request. A reporting summary for this Article is available as a Supplementary Information file.
