# Supplementary material for: Lsd1 as a therapeutic target in Gfi1-activated medulloblastoma
Source: Nat Commun. 2019 Jan 18;10:332. doi: 10.1038/s41467-018-08269-5 (PMC6338772; doi:10.1038/s41467-018-08269-5)
Supplement: Supplementary file 1 — Supplementary Information [file 41467_2018_8269_MOESM1_ESM.pdf]

Supplementary Information

Lsd1 as a therapeutic target in Gfi1-activated medulloblastoma.

Lee et al.

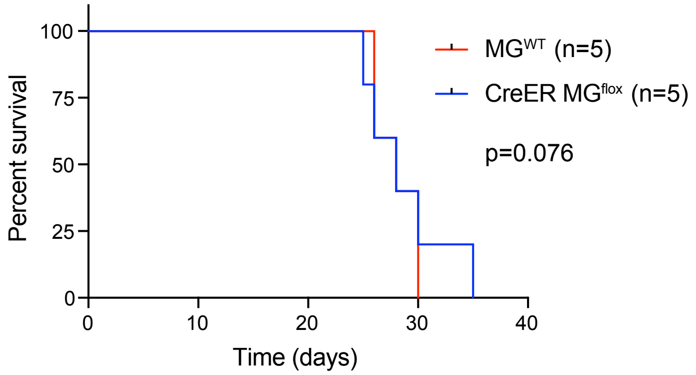

Supplementary Figure 1. MG^{flox} and MG^{WT} tumors exhibit similar latency and penetrance.

Survival curves comparing mice with MG^{flox} (n=5) and MG^{WT} (n=5) tumors. Median survival is 28 days for both types of tumors. p value=0.076 (not significant) determined by Log-rank (Mantel-Cox) test. Data shown are from a representative experiment.

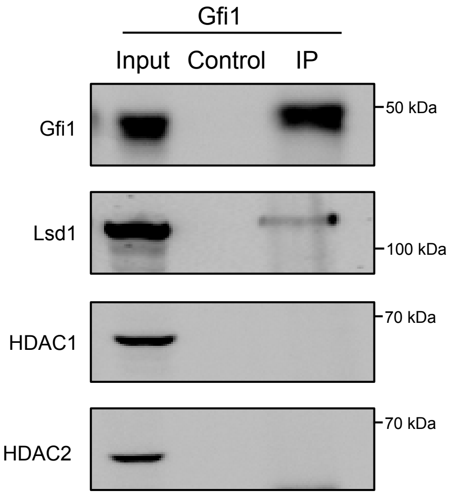

Supplementary Figure 2. Gfi1 is not associated with HDAC1 or HDAC2.

Co-immunoprecipitation of Gfi1 was performed on MG tumor cells, and levels of Gfi1, Lsd1, HDAC1, and HDAC2 were determined by Western blotting. Input represents 10% of total lysate before immunoprecipitation with experimental and isotype control antibodies. Data shown are from a representative experiment, where experiments were repeated in 3 biological replicates.

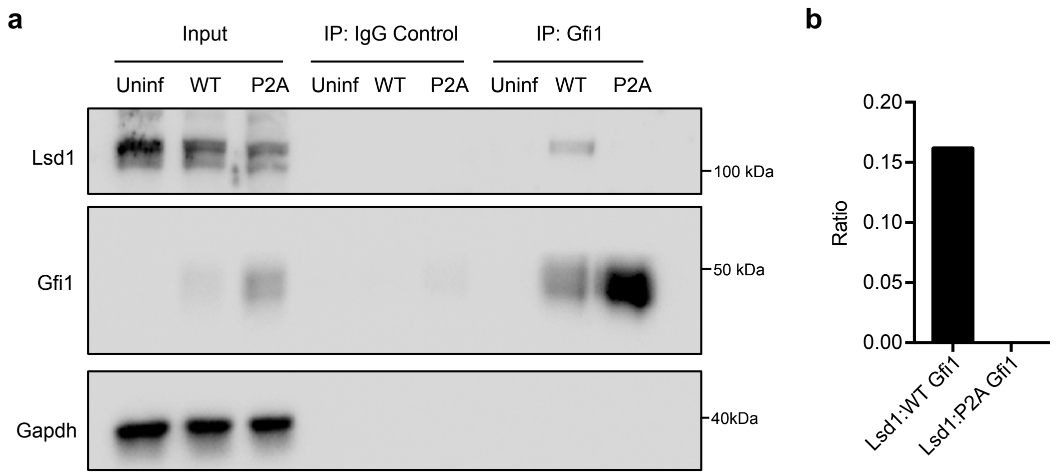

Supplementary Figure 3. The Gfi1-P2A SNAG domain mutant cannot recruit and bind Lsd1.

a Co-immunoprecipitation of Gfi1 was performed on 3T3 cells infected with wildtype Gfi1 (WT) or SNAG mutant Gfi1 (P2A), and levels of Gfi1 and Lsd1 were determined by Western blotting. Input represents 10% of total lysate before immunoprecipitation with experimental and isotype control antibodies. **b** Quantification of Western blot indicating the ratio of Lsd1 pulled down to Gfi1 pulled down. Data shown are from a representative experiment, where experiments were repeated in 3 biological replicates.

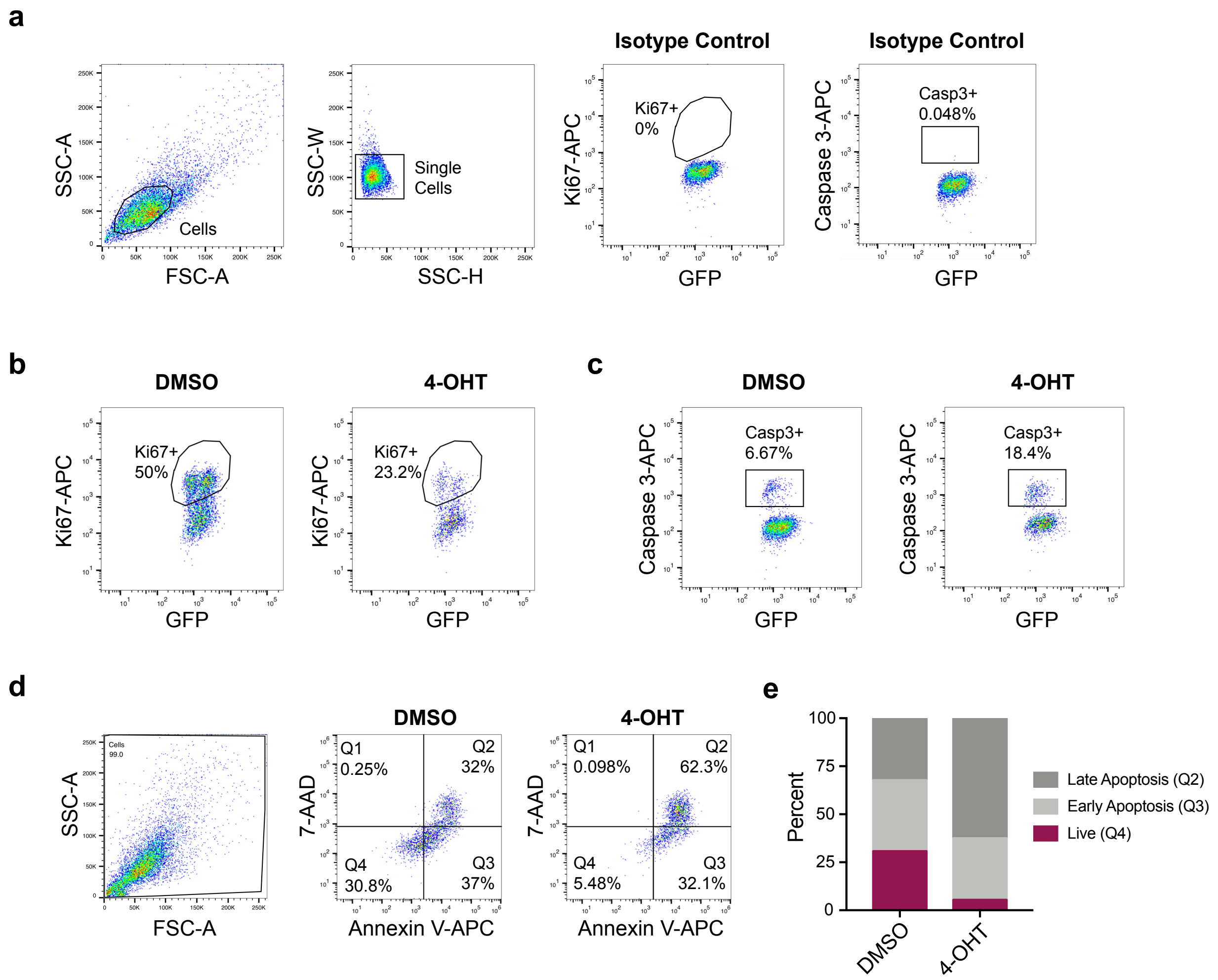

Supplementary Figure 4. Deletion of Lsd1 in MG tumor cells decreases proliferation and increases cell death.

a-c Lsd1 iKO MG tumor cells were treated overnight with vehicle (DMSO) or 5 μM 4-hydroxytamoxifen (4-OHT). After ~40 hours, cells were fixed and stained with **(a)** isotype control, **(b)** Ki67 antibody or **(c)** active Caspase 3 antibody. **a** Left two plots show the gating strategy for cells using FSC and SSC characteristics. Right plots show the gating strategy for Ki67+ and Caspase 3+ cells as determined by staining samples with an isotype control antibody. **b** Representative dot plots for Ki67 staining in cells treated with DMSO or 5 μM 4-OHT. Deletion of Lsd1 caused a decrease in proliferation as 50% of

DMSO-treated cells are Ki67+, while only 23.2% of 4-OHT-treated cells are Ki67+. **b** Representative dot plots for active Caspase 3 staining in cells treated with DMSO or 5 μM 4-OHT. 6.67% of DMSO-treated cells are Caspase 3+, while 18.4% of 4-OHT-treated cells are Caspase3+. **d, e** Lsd1 iKO MG tumor cells were treated overnight with DMSO or 5 μM 4-OHT. After ~40 hours, cells were stained with Annexin V antibody and 7-AAD. **d** Representative dot plots for Annexin V and 7-AAD staining in cells treated with DMSO or 5 μM 4-OHT. Left plot shows the gating strategy for cells using FSC and SSC characteristics. **e** Quantification of live cells and cells going through apoptosis as seen in panel **d**. DMSO-treated cells consist of ~30% live cells and ~70% dying cells. 4-OHT-treated cells consist of ~6% live cells and ~94% dying cells. The data shown are from representative experiments, where all staining experiments were repeated in at least 3 biological replicates.

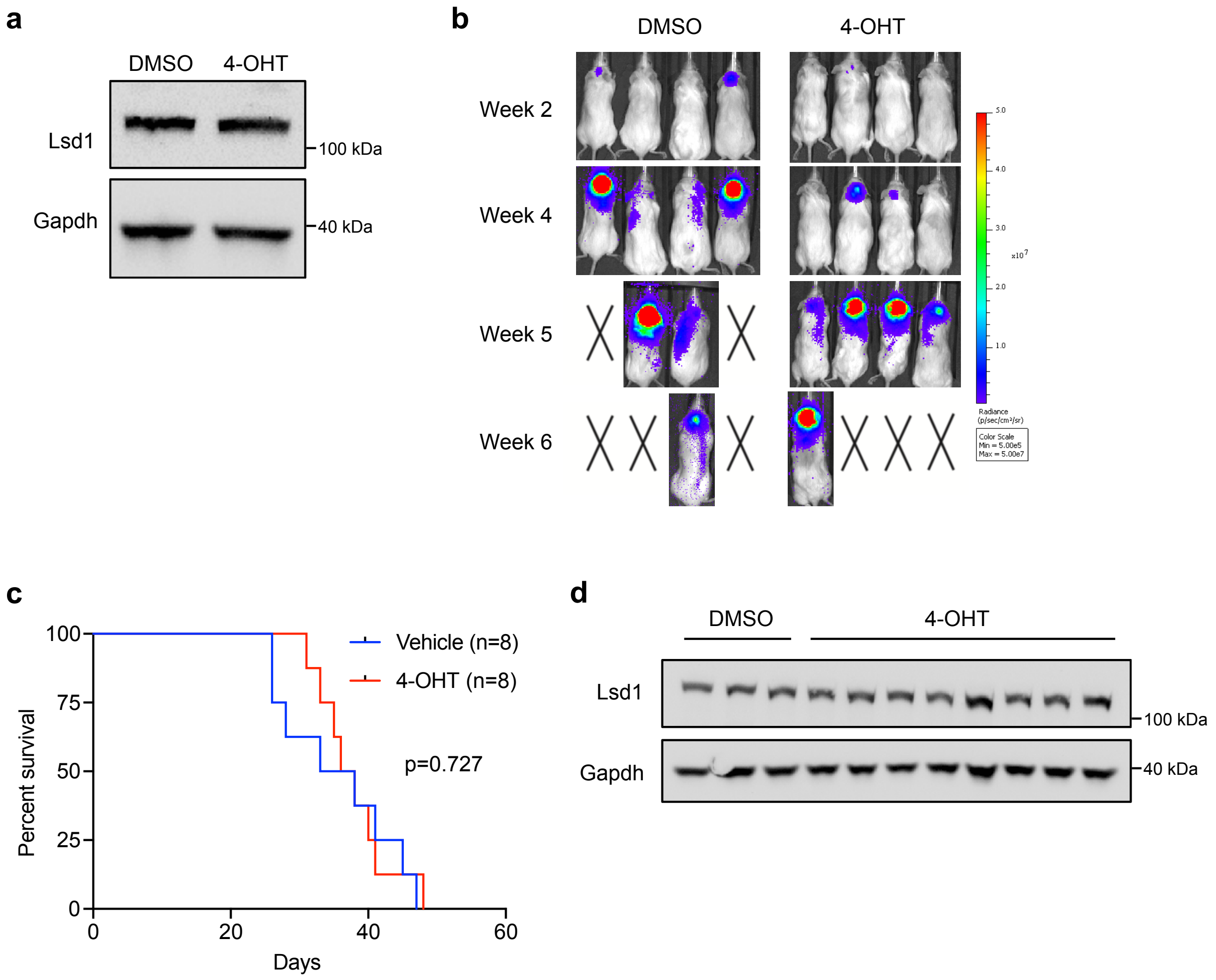

Supplementary Figure 5. Treatment with 4-OHT does not directly affect MG tumor growth.

a Western blot for Lsd1 in Lsd1$^{fl/fl}$ MG tumor cells that were treated overnight with vehicle control (DMSO) or 5 μM 4-hydroxytamoxifen (4-OHT). **b** Bioluminescence imaging of mice transplanted with Lsd1$^{fl/fl}$ MG cells treated with vehicle or 4-OHT. X's denote animals euthanized before they could be imaged. **c** Survival curves from a representative experiment (control n=8, 4-OHT n=8). p value=0.7271 was determined by Log-rank (Mantel-Cox) test. **d** Western blot for Lsd1 protein levels in resulting tumors from both

vehicle and 4-OHT treatment groups. Data shown are from a representative experiment, where experiments were repeated in 3 biological replicates.

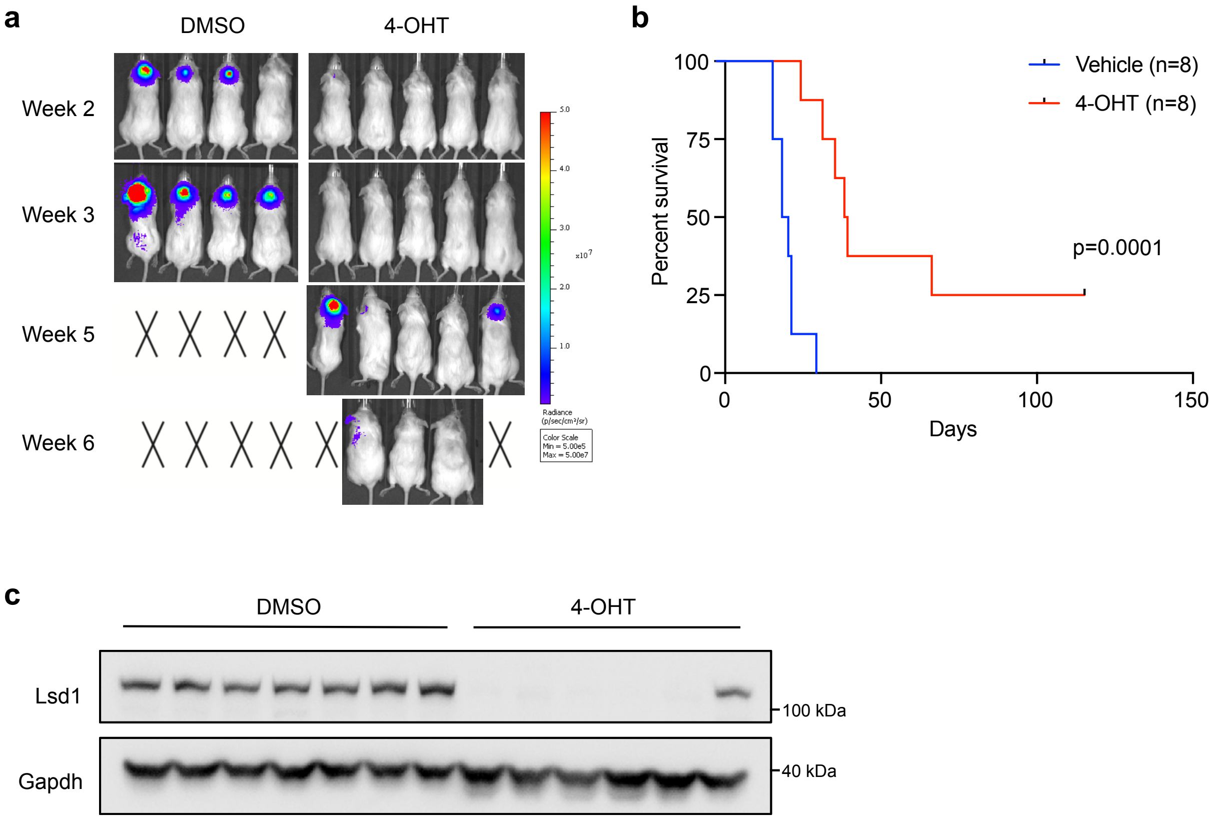

Supplementary Figure 6. Lsd1 is not required for MP tumor growth.

Lsd1-iKO Myc+DNp53 (MP) tumor cells were treated with vehicle control (DMSO) or 5 μM 4-hydroxytamoxifen (4-OHT) and transplanted into mice the following day. **a** Bioluminescence imaging of mice transplanted with Lsd1-iKO MP cells treated with vehicle or 4-OHT. X's denote animals euthanized before they could be imaged. **b** Survival curves from a representative experiment (control n=8, 4-OHT n=8). p value=0.0001 was determined by Log-rank (Mantel-Cox) test. **c** Western blot for Lsd1 protein levels in resulting tumors from both vehicle and 4-OHT treatment groups. Data shown are from a representative experiment, where experiments were repeated in 3 biological replicates.

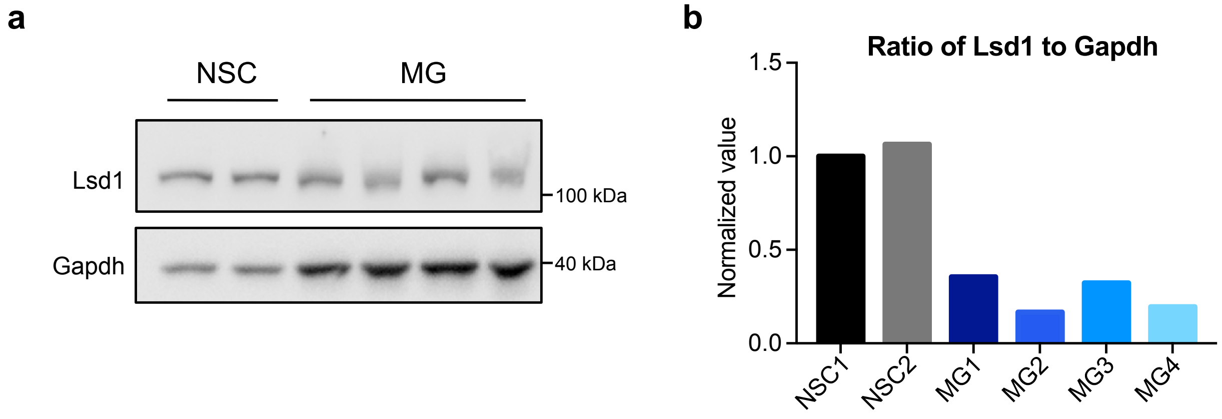

Supplementary Figure 7. Lsd1 expression in MG tumors is normal compared to NSCs and MP tumors.

a Western blot comparing Lsd1 protein levels in NSCs and MG tumor cells. **b** Quantification of Western blot. Plotted values represent the ratio of Lsd1 to Gapdh, relative to that of the NSC1 sample. Lsd1 expression in MG tumors is not higher than the levels found in NSCs.

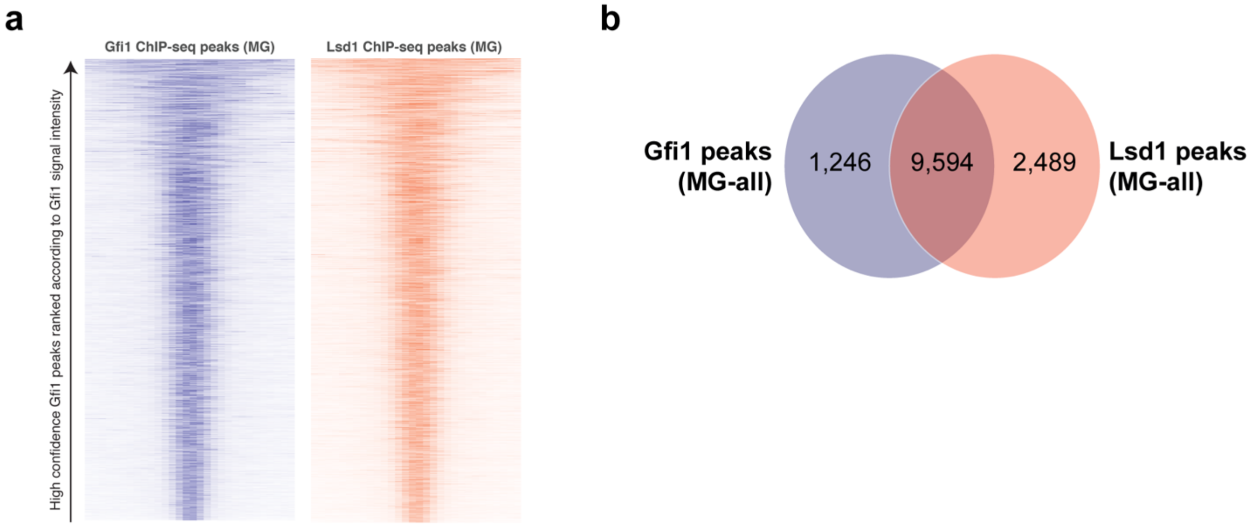

Supplementary Figure 8. Gfi1 and Lsd1 co-occupy many of the same genes in MG tumor cells.

ChIP-seq for Gfi1 and Lsd1 was conducted in MG tumor cells to determine binding in the genome. **a** Heat maps show the scaled read densities surrounding ± 3kb midpoint Gfi1 peaks for Gfi1 and Lsd1. Only high confidence (see Methods) Gfi1 peaks are shown and ordered according to the Gfi1 signal intensity. **b** Venn diagram showing the overlap between high confidence Gfi1 and Lsd1 peaks.

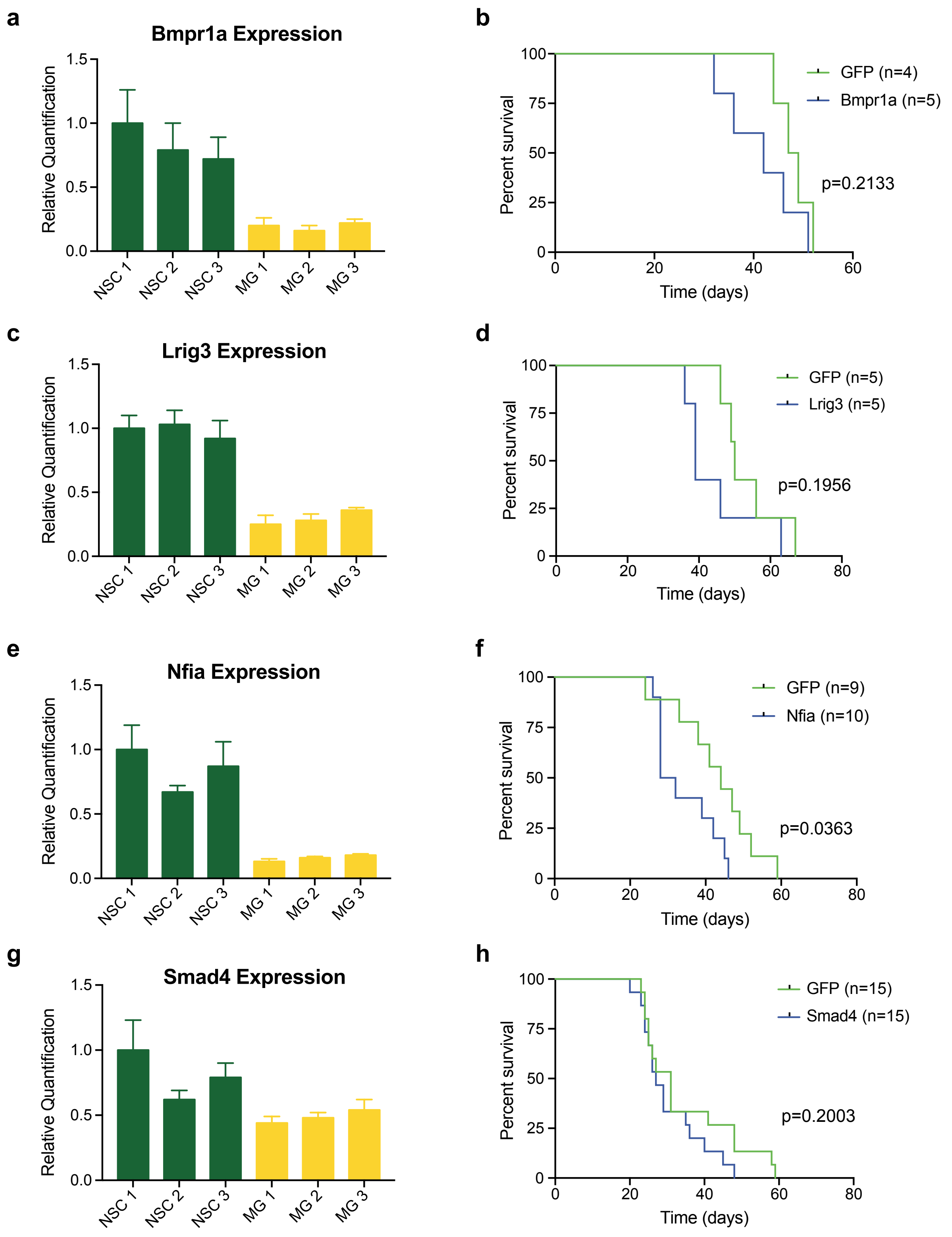

Supplementary Figure 9. ***In vivo*** **functional assessment of candidate Gfi1 target genes.**

Candidate Gfi1 target genes known to be involved in differentiation processes were expression validated by qPCR and then functionally validated using overexpression studies. qPCR results comparing **(a)** Bmpr1a, **(c)** Lrig3, **(e)** Nfia, and **(g)** Smad4 expression in biological triplicates of NSCs and MG tumor cells confirm that these genes are downregulated in MG tumors. Error bars represent 95% confidence intervals calculated using the sum of squares method. To evaluate the functional significance of these genes in MG tumors, tumor cells were infected with empty vector (GFP) or with the genes of interest, sorted, and re-transplanted into mice. Resulting survival curves for **(b)** Bmpr1a (control n=4, Bmpr1a n=5, p=0.2133), **(d)** Lrig3 (control n=5, Lrig3 n=5, p=0.1956), **(f)** Nfia (control n=9, Nfia n=10, p=0.0363), and **(h)** Smad4 (control n=15, Smad4 n=15, p=0.2003) indicate no survival advantage upon overexpression in MG tumors. p values were determined by Log-rank (Mantel-Cox) test.

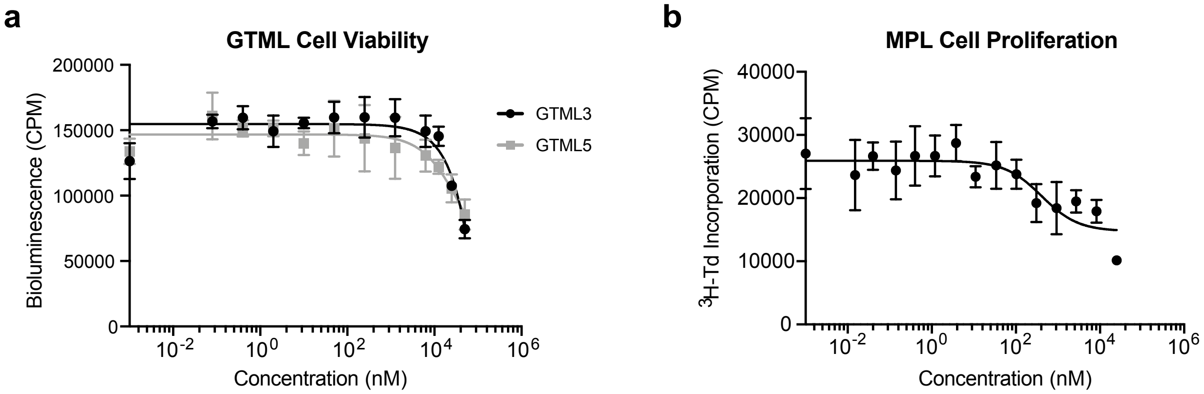

Supplementary Figure 10. Pharmacological inhibition of Lsd1 is less effective on non-Gfi1-activated tumors.

a Cell viability of two different GTML tumor cell lines treated with GSK-LSD1. Viability was measured via CellTiter-Glo Luminescent Assay after 48hrs of drug treatment. Data are from a representative experiment and are plotted as the means of technical triplicate samples ± SEM. **b** In vitro proliferation assay for the SHH subtype MPL tumor cells treated with GSK-LSD1. Proliferation was measured via ^{3}H-thymidine incorporation after 48hrs of drug treatment. Data are from a representative experiment and are plotted as the means of technical triplicate samples ± SEM. All experiments were repeated in at least 3 biological replicates. The viability of GTML and proliferation of MPL tumor cells were not fully inhibited by treatment with GSK-LSD1, so accurate IC_{50}s could not be calculated.

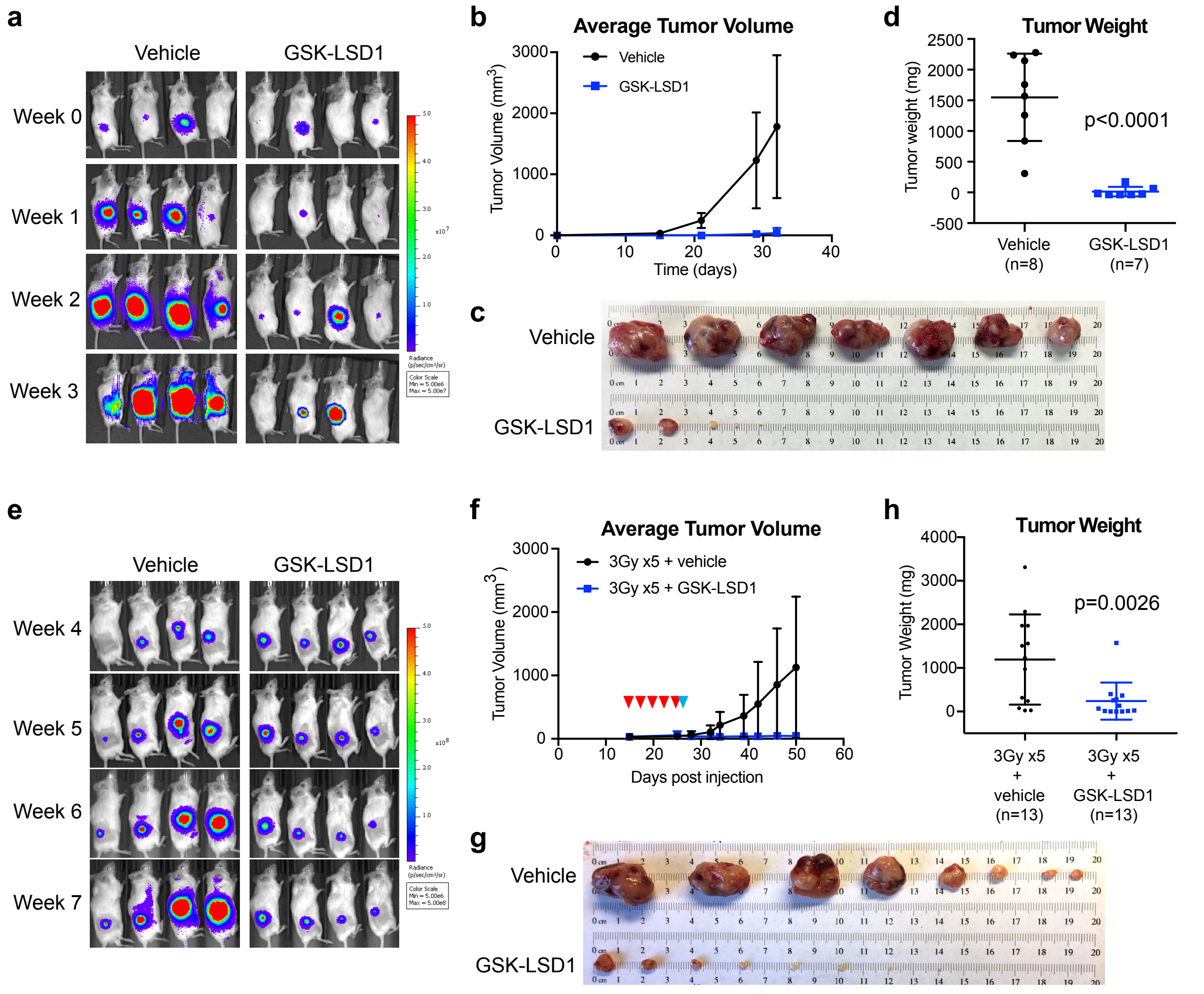

Supplementary Figure 11. Pharmacological inhibition of Lsd1 and radiotherapy inhibit MG tumor growth.

a-d Mice implanted with subcutaneous MG tumors were treated with vehicle (4% DMSO in saline) or 10 mg/kg GSK-LSD1 via i.p. injection in cycles of four days on and three days off (vehicle n=8, GSK-LSD1 n=7). Tumor growth was monitored weekly by **(a)** bioluminescence imaging and **(b)** caliper measurements. When tumors reached the maximum allowed diameter of 2 cm, mice were sacrificed and resulting tumors were **(c)** removed and **(d)** weighed. GSK-LSD1 treatment significantly reduced tumor burden

(p<0.0001, t=5.661, df=13, one-tailed unpaired t-test) in mice. Data shown are from a representative experiment, where experiments have been repeated in 3 biological replicates. **e-h** Mice implanted with subcutaneous MG tumors were irradiated with 5 doses of 3 Gy and treated with vehicle (saline) or 10 mg/kg GSK-LSD1 in cycles of four days on and three days off (vehicle n=13, GSK-LSD1 n=13). Tumor growth was monitored weekly by **(e)** bioluminescent imaging and **(f)** caliper measurements. Red arrowheads indicate time of irradiation. Blue arrowhead indicates the start of drug treatment. When tumors reached the maximum allowed diameter, mice were sacrificed and resulting tumors were **(g)** collected and **(h)** weighed. Compared to radiation therapy alone, the combination of radiation and GSK-LSD1 treatment significantly reduced tumor burden (p=0.0026, t=3.07, df=24, one-tailed unpaired t-test) in mice. Data shown in (**h**) are from 2 replicate experiments. Average tumor volume over time is plotted as the means ± SEM, and final tumor weights are plotted as the means ± SD.

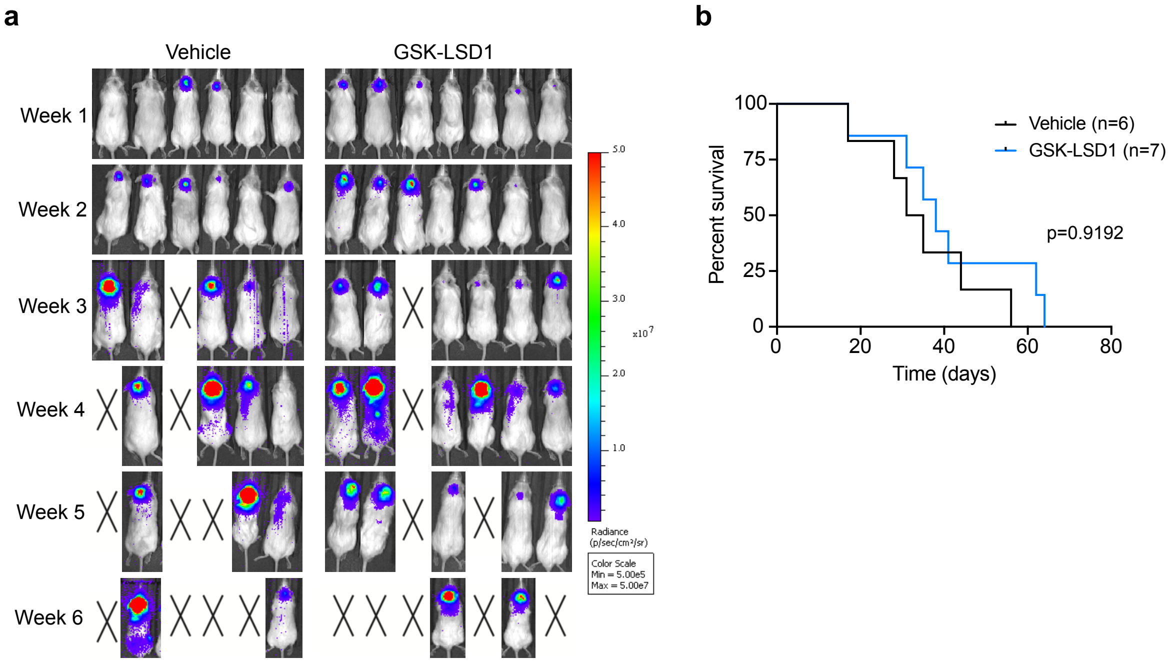

Supplementary Figure 12. Treatment with Lsd1 inhibitor does not significantly inhibit the growth of intracranial MG tumors.

a, b Mice implanted with intracranial MG tumors were treated with vehicle (4% DMSO in saline) or 10 mg/kg GSK-LSD1 via i.p. injection in cycles of four days on and three days off (vehicle n=8, GSK-LSD1 n=7). **a** Tumor growth was monitored weekly by bioluminescence imaging. **b** Survival curves for mice treated with vehicle and mice treated with GSK-LSD1 showed no significant difference. p value=0.9192 (not significant) determined by Log-rank (Mantex-Cox) test. Data shown are from one representative experiment.
