# Supplementary material for: Lsd1 as a therapeutic target in Gfi1-activated medulloblastoma
Source: Nat Commun. 2019 Jan 18;10:332. doi: 10.1038/s41467-018-08269-5 (PMC6338772; doi:10.1038/s41467-018-08269-5)
Supplement: Supplementary file 2 — Description of Additional Supplementary Files [file 41467_2018_8269_MOESM2_ESM.docx]

**Description of Supplementary Files**

**File Name:** Supplementary Data 1

**Description:** Differentially expressed genes in MG tumor cells compared to NSCs. Gene expression data for n=7 MG and n=5 NSC samples were analyzed for 21,304 unique genes. Expression values were corrected, normalized, and transformed to log base 2 scale. Differentially expressed genes were defined using a cut-off of absolute value of the log fold change (>/=1.5) between average expression values in MG versus NSC samples and with corrected p-values of </= 10^-2 (n=2,402 genes).

**File Name:** Supplementary Data 2

**Description:** Significant Gfi1 and Lsd1 ChIP-seq peaks in MG tumors. ChIP-seq for Gfi1 and Lsd1 was conducted in MG tumor cells to determine their genome-wide occupancies. Using MACS with default parameters, we identified 10,840 significant peak regions bound by Gfi1 and 12,083 peak regions bound by Lsd1, with 9,594 peaks bound by both Gfi1 and Lsd1.
