# Supplementary material for: Lsd1 as a therapeutic target in Gfi1-activated medulloblastoma
Source: Nat Commun. 2019 Jan 18;10:332. doi: 10.1038/s41467-018-08269-5 (PMC6338772; doi:10.1038/s41467-018-08269-5)
Supplement: Supplementary file 5 — Reporting Summary [file 41467_2018_8269_MOESM5_ESM.pdf]

nature research

Corresponding author(s): *Double-blind peer review submissions: write DBPR and your manuscript number here instead of author names.*

Reporting Summary

Nature Research wishes to improve the reproducibility of the work that we publish. This form provides structure for consistency and transparency in reporting. For further information on Nature Research policies, see Authors & Referees and the Editorial Policy Checklist.

Statistical parameters

When statistical analyses are reported, confirm that the following items are present in the relevant location (e.g. figure legend, table legend, main text, or Methods section).

n/a	Confirmed	
☐	☒	The exact sample size (*n*) for each experimental group/condition, given as a discrete number and unit of measurement
☐	☒	An indication of whether measurements were taken from distinct samples or whether the same sample was measured repeatedly
☐	☒	The statistical test(s) used AND whether they are one- or two-sided<br>*Only common tests should be described solely by name; describe more complex techniques in the Methods section.*
☒	☐	A description of all covariates tested
☐	☒	A description of any assumptions or corrections, such as tests of normality and adjustment for multiple comparisons
☐	☒	A full description of the statistics including central tendency (e.g. means) or other basic estimates (e.g. regression coefficient) AND variation (e.g. standard deviation) or associated estimates of uncertainty (e.g. confidence intervals)
☐	☒	For null hypothesis testing, the test statistic (e.g. *F*, *t*, *r*) with confidence intervals, effect sizes, degrees of freedom and *P* value noted<br>*Give P values as exact values whenever suitable.*
☒	☐	For Bayesian analysis, information on the choice of priors and Markov chain Monte Carlo settings
☒	☐	For hierarchical and complex designs, identification of the appropriate level for tests and full reporting of outcomes
☒	☐	Estimates of effect sizes (e.g. Cohen's *d*, Pearson's *r*), indicating how they were calculated
☐	☒	Clearly defined error bars<br>*State explicitly what error bars represent (e.g. SD, SE, CI)*

Our web collection on statistics for biologists may be useful.

Software and code

Policy information about availability of computer code

Data collection	No software was used to collect data.
Data analysis	Affymetrix data was pre-processed using the RMA algorithm. Differential gene expression was determined using the "limma" package in R, and the BH method was used for multiple testing correction. Pathway analysis of the differentially expressed genes was performed using the ClueGO plug-in for Cytoscape.<br><br>ChIP-sequencing reads were aligned to the mouse reference genome (mm10, chromosomes 1-19, X, Y and M) using the Burrow-Wheeler Transformation based aligner "BWA," version 0.6.2, arguments -q20. BAM files were merged using Picard (http://picard.sourceforge.net). Peak calling was done using MACS, and gene annotations were based on gencode vM9 (http://www.gencodegenes.org/mouse_releases/0.html).

For manuscripts utilizing custom algorithms or software that are central to the research but not yet described in published literature, software must be made available to editors/reviewers upon request. We strongly encourage code deposition in a community repository (e.g. GitHub). See the Nature Research guidelines for submitting code & software for further information.

Data

Policy information about availability of data

All manuscripts must include a data availability statement. This statement should provide the following information, where applicable:

- Accession codes, unique identifiers, or web links for publicly available datasets
- A list of figures that have associated raw data
- A description of any restrictions on data availability

Figure 6, Supplemental Figure 8, Supplemental Table 1, and Supplemental Table 2 have associated raw data (Affymetrix and ChIP-seq) ...

Field-specific reporting

Please select the best fit for your research. If you are not sure, read the appropriate sections before making your selection.

☒ Life sciences ☐ Behavioural & social sciences ☐ Ecological, evolutionary & environmental sciences

For a reference copy of the document with all sections, see nature.com/authors/policies/ReportingSummary-flat.pdf

Life sciences study design

All studies must disclose on these points even when the disclosure is negative.

Sample size	Based on previous experience with studies of this type, power analysis indicates 8 mice per group will have 90% power to detect an average survival difference of 14 days with a significance of p = 0.05. Thus, most experiments included at least 8 mice/group and were repeated at least 3 times. In some cases where limited amounts of cells were available for transplant into mice, fewer than than 8 mice were used and the sample size is indicated as such.
Data exclusions	For survival studies evaluating tumorigenesis, no data were excluded from analysis. For drug treatment studies on subcutaneous flank tumors, a few mice were excluded because they died prematurely before the experiment endpoint (ie. death due to circumstances unrelated to tumor burden). Mice excluded from analysis were not included in reported sample sizes.
Replication	Experiments were done at least three times. Attempts at replication were successful.
Randomization	For in vivo treatment experiments, tumor-bearing mice were subjected to bioluminescent imaging and caliper measurements. Animals with comparable tumor sizes were randomized into treatment groups; this prevented outcomes from being influenced by initial differences in tumor burden.
Blinding	Investigators were not blinded to group allocation.

Reporting for specific materials, systems and methods

Materials & experimental systems

n/a	Involved in the study
☐	☒ Unique biological materials
☐	☒ Antibodies
☐	☒ Eukaryotic cell lines
☒	☐ Palaeontology
☐	☒ Animals and other organisms
☒	☐ Human research participants

Methods

n/a	Involved in the study
☐	☒ ChIP-seq
☐	☒ Flow cytometry
☒	☐ MRI-based neuroimaging

Unique biological materials

Policy information about availability of materials

Obtaining unique materials	Our study uses primary murine medulloblastoma cells, which will be made available to other investigators upon request. Established cell lines (293T and NIH-3T3) are commercially available through ATCC.

Antibodies

Antibodies used	Details about all antibodies used in this study are provided in the Materials and Methods section of the manuscript.
Validation	Validation statements for antibodies used for immunoprecipitation (Gfi1, Lsd1, CoREST), Western blot (Gapdh, Actin, Gfi1, Lsd1, CoREST, HDAC1, HDAC2, p53, p21), and flow cytometry (Ki67, Caspase 3, Annexin V) can be found on their corresponding manufacturer websites. Validation in our own samples has been confirmed by Western blot detection of bands at the predicted size.

Eukaryotic cell lines

Policy information about cell lines

Cell line source(s)	NIH-3T3 and HEK 293T/17 were obtained from ATCC.
Authentication	Cell lines have been authenticated by ATCC.
Mycoplasma contamination	Authors declare that cell lines used were not tested for mycoplasma contamination.
Commonly misidentified lines (See ICLAC register)	N/A

Animals and other organisms

Policy information about studies involving animals; ARRIVE guidelines recommended for reporting animal research

Laboratory animals	Animal strains used for the study are described in Methods section. Male and female NSG mice are used as hosts for transplantation at 6-7 weeks of age. 5-7 day old pups from C57/BL6, CAG-CreER, and CAG-CreER Lsd1 flox mice were used to generate neural stem cells, which were then transduced with oncogenes to generate primary mouse medulloblastoma.
Wild animals	This study did not involve wild animals.
Field-collected samples	This study did not involve field-collected samples.

ChIP-seq

Data deposition

☒ Confirm that both raw and final processed data have been deposited in a public database such as GEO.

☒ Confirm that you have deposited or provided access to graph files (e.g. BED files) for the called peaks.

Data access links<br>*May remain private before publication.*	https://www.ncbi.nlm.nih.gov/geo/query/acc.cgi?acc=GSE123870
Files in database submission	tumor_WT_39_GFI1_GGAGCTA_merged.bam.rmdup.bam<br>tumor_WT_41_GFI1_TGCAGAG_merged.bam.rmdup.bam<br>tumor_WT_39_LSD1_GATTCGC_merged.bam.rmdup.bam<br>tumor_WT_41_LSD1_CCTTGTC_merged.bam.rmdup.bam<br>tumor_Input-MGB-Pool_ACAGTG_merged.bam.rmdup.bam<br>tumor_WT_39_GFI1_GGAGCTA_merged.bam.rmdup.tdf<br>tumor_WT_41_GFI1_TGCAGAG_merged.bam.rmdup.tdf<br>tumor_WT_39_LSD1_GATTCGC_merged.bam.rmdup.tdf<br>tumor_WT_41_LSD1_CCTTGTC_merged.bam.rmdup.tdf<br>tumor_Input-MGB-Pool_ACAGTG_merged.bam.rmdup.tdf<br>WT_39_GFI1_GGAGCTA_peaks_peaks.bed<br>WT_41_GFI1_TGCAGAG_peaks_peaks.bed<br>WT_39_LSD1_GATTCGC_peaks_peaks.bed<br>WT_41_LSD1_CCTTGTC_peaks_peaks.bed
Genome browser session<br>(e.g. UCSC)	No longer applicable.

Methodology

Replicates	Biological replicates (MG_tumor_WT_39 & MG_tumor_WT_41) were subjected to both Gfi1 and Lsd1 ChIP.
Sequencing depth	Sample Number of reads mapped Number of uniquely mapped reads Mean coverage Read length Type<br>MGB-Pool_ACAGTG 92556506 85621577 5.90465 100bp paired-end<br>WT_39_GFI1_GGAGCTA 60302626 57191064 4.79595 100bp paired-end<br>WT_41_GFI1_TGCAGAG 85149793 80718696 6.76885 100bp paired-end

	WT_39_LSD1_GATTCGC 94738309 90406096 7.53462 100bp paired-end<br>WT_41_LSD1_CCTTGTC 57322001 54465071 4.5608 100bp paired-end
Antibodies	Gfi1: Rabbit polyclonal, anti-Gfi1 antibody, GNE-CJS-2 (4ug) obtained directly from H. Leighton Grimes.<br>Lsd1: Rabbit polyclonal, anti-Lsd1, Abcam #ab17721 (4ug).
Peak calling parameters	MACS was used with default parameters.
Data quality	Sequence quality was assessed by FastQC (https://www.bioinformatics.babraham.ac.uk/projects/fastqc/).
Software	Not applicable.

Flow Cytometry

Plots

Confirm that:

☒ The axis labels state the marker and fluorochrome used (e.g. CD4-FITC).

☒ The axis scales are clearly visible. Include numbers along axes only for bottom left plot of group (a 'group' is an analysis of identical markers).

☒ All plots are contour plots with outliers or pseudocolor plots.

☒ A numerical value for number of cells or percentage (with statistics) is provided.

Methodology

Sample preparation	Isolation of tumor cells from mice is described in the Methods section of the manuscript. Samples prepared after in vitro culture were briefly treated with trypsin to dissociate cells into single cell suspension for flow cytometric analysis and sorting.
Instrument	BD FACSAriaIII (cat#648282-01, year 2010)
Software	Data were acquired using FACSDiva and analyzed using FlowJo.
Cell population abundance	For over-expression studies, tumor cells were infected with vectors containing a GFP reporter. GFP+ expression, and abundance varied across samples due to differences in infection efficiency. Post-sort purity was confirmed by analyzing aliquots of the sorted cells.
Gating strategy	For Ki67 and Caspase 3 analysis, tumor cell populations were first gated to exclude cell debris and aggregates based on FSC/SSC. Then cells stained with isotype control were used to determine the boundary between "negative" and "positive" cells; this boundary was used to identify positive cells in samples stained with the specific antibody. For Annexin V analyses, FSC/SSC gates were only very loosely applied to include most events.

☒ Tick this box to confirm that a figure exemplifying the gating strategy is provided in the Supplementary Information.
